# Distinct types of VHHs in Alpaca

**DOI:** 10.3389/fimmu.2024.1447212

**Published:** 2024-11-12

**Authors:** Xinhao Wang, Lu Zhang, Yao Zhang, Jiaguo Li, Wenfeng Xu, Weimin Zhu

**Affiliations:** ^1^ Drug Discovery and Development, Chantibody Therapeutics, Menlo Park, CA, United States; ^2^ Drug Discovery and Development, Shanghai Cell Therapy Group Co. Ltd, Shanghai, China

**Keywords:** VHH, VHH-Ag interaction, epitope, paratope, nanobody, single-domain antibody

## Abstract

**Introduction:**

VHHs (VH of heavy-chain-only antibodies) represent a unique alternative to Q7 conventional antibodies because of their smaller size, comparable binding affinity and biophysical properties.

**Method:**

In this study, we systematically analyzed VHH NGS sequences from 22 Alpacas and structure data from public database.

**Results:**

VHHs in Alpaca can be grouped into five main types with multiple distinct sequence and structure features. Based on the existence of hallmark residues in FR2 region, VHHs can be classified into two groups: nonclassical VHHs (without hallmark residues) and classical VHHs (with hallmark residues). Based on VHH hallmark residues at 42 position (IMGT numbering, FR2 region) and number of cysteines, we found that Alpaca classical VHHs can be further separated into three main types: F_C2 VHHs with F (phenylalanine) at position 42 and having 2 cysteines within sequences, Y_C2 VHHs with Y (tyrosine) at position 42 and having 2 cysteines, and F_C4 with F at position 42 and having 4 cysteines. Non-classical VHHs can be further separated into 2 types based on germlines mapped: N_V3 for VHHs mapped to V3 germlines and N_V4 for V4 germlines. Based on whether FR2 residues are involved in binding, two kinds of paratopes can be identified. Different types of VHHs showed distinct associations with these two paratopes and displayed significant differences in paratope size, residue usage and other structure features.

**Discussion:**

Such results will have significant implications in VHH discovery, engine e ring, and design for innovative therapeutics.

## 1 Introduction

Heavy-chain-only antibodies (HCAbs) exist naturally in the immune repertoire of camelids and cartilaginous fish (1). HCAbs with homodimer form consist of one variable region (VHH) and two constant domains. VHH, sufficient for antigen binding, has dimensions in the nanometer range with about 15kD molecular weight. It is also known as a nanobody because of its nanometer size or single-domain antibody (sdAb). VHHs have been extensively studied because of their many applications as research reagents, diagnostic tools and therapeutic drugs (2–4). It has many properties uniquely different from conventional antibodies: small size, high affinity and specificity, better solubility and thermostability, ability to target special epitopes like cavities etc.

Previous work (1, 5) has identified several hallmark residues at positions 42, 49, 50, 52 (IMGT numbering) in the FR2 region of VHH sequences. They are distinctly different from conventional heavy chains: Val42 → Tyr/Phe, Gly49 → Glu, Leu50 → Arg, and Trp52 → Leu/Gly/Phe. These hallmark residues are considered to play important roles in stabilizing VHHs in the absence of light chains (6). However, not all VHHs have these hallmark residues. Based on whether these hallmark residues exist or not, VHHs can be grouped into classical VHHs (with hallmark residues) and non-classical VHHs (without hallmark residues). For non-classical VHHs, some of them have Trp in the beginning of FR4 sequence replaced by Arg, possibly another mechanism to stabilize VHHs (7, 8).

Next generation sequencing (NGS) technology provides an effective tool to analyze various properties of whole immune repertoire by sequencing millions of antibody sequences with high efficiency and low cost. Using NGS technology, Li et al. (9) compared the repertoires of conventional antibodies and VHHs of Bactrian camels, and found significant longer CDR3 and higher somatic hyper mutation (SHM) in VHHs. Henry et al. (10) studied conventional and HCAb IgG subtypes in Llama peripheral B-cell populations, and found similar results. In addition, they found a low percentage of hingeless HCAbs in immune repertoire. More recently, Tu et al. (11) systematically analyzed VHH CDR3 length distribution, VDJ usage, germline-specific mutation and other properties using VHH NGS sequences generated from peripheral blood mononuclear cells (PBMCs) of multiple male Alpacas.

Using published structure data, extensive structure comparative analyses (12–14) between conventional antibodies and VHHs have been performed and showed several unique characteristics of VHHs: more likely to involve framework residues in paratope, smaller paratope but similar epitope size, etc. Murakami et al. (15) proposed a classification for paratope formation as either upright, half-roll or roll. Dizicheh et al. (16) found two main VHH CDR3 conformations, extended and kinked, depending on the germlines they are from. They also showed the importance of FR2 residues in maintaining such CDR3 conformation. However, they did not further analyze distinct differences in sequence and structure features of VHHs from these germlines.

In this work, we performed systemic analysis on NGS sequences from PBMC of 22 Alpacas and VHH structure data from public database and found that there are 5 main types of VHHs in Alpaca with distinct sequence and structure differences among these types. Such findings provide a better understanding of different types of VHHs in immune repertoire, sequence, structure, and function relationships, and will be valuable in VHH discovery, engineering, synthetic library design and therapeutics development.

## 2 Methods and materials

### 2.1 VHH sequencing and analysis.

PBMCs from 22 Alpacas (**Supplementary Table S1**) before immunization were collected and NGS libraries were built using primers targeting VHH hinge region and leader sequences (**Supplementary Table S2**). Libraries were sequenced using MiSeq (Illumina, Inc) with 2x300 PE module. Sequences were processed using internally developed bioinformatics workflow to identify CDR1/CDR2/CDR3 and framework regions based on IMGT numbering (17), and biophysical-chemical properties of each VHH sequence were analyzed. To identify possible germline for each VHH sequence and estimate the SHM rate, VHH sequences were aligned with Alpaca germlines downloaded from IMGT (18) using blastn (19) with similar parameters as used in Igblast (20). The average number of mismatches in 100 bp alignment was used to estimate SHM rate. The net charges of the different VHH regions at pH 7.4 were calculated by summing the charges of D (−1), E (−1), R (+1), K (+1) and H (+0.1). VHH Isoelectric point (PI) was calculated using IPC tool (21). Hydropathy indices for different VHH regions were calculated by averaging the hydropathy index (22) of each residue within the region. To minimize errors introduced during PCR and sequencing steps, only sequences with at least 5 counts were used. Duplicated sequences were removed to ensure each sequence in the set was unique at the amino acid level. A total of 467562 VHH sequences were used in the analysis.

### 2.2 Uniform Manifold Approximation and Projection analysis

AntiBERTy (23), nanoBERT (24) and ESM2 (25) language models were used to generate embeddings for the sequences. For AntiBERTy model, we used “embed” method as recommended by the author to generate embeddings. For the other two models, we extracted values from the last layer. Per residue embeddings were further averaged along the length of input sequence and resulted vectors were used as input for UMAP analysis.

### 2.3 Structure dataset and analysis

Crystal structures of antigen-VHH complexes were extracted from SAbDab-nano (26) on Aug 1st, 2023. Data was further processed as follows: Firstly, only complexes with protein antigens and species labeled as originating from llama, alpaca, camel, or vicugna pacos were retained. Secondly, all the complexes whose epitopes with less than 8 amino acids were removed to exclude potential false interactions. Thirdly, de-redundancy was performed based on VHH sequence identity, which resulted in a non-redundancy structural dataset consisting of 520 antigen-VHH complexes.

VHH structure data in the database are from different species including Alpaca, Llama, Bactrian, and Dromedary with more than 50% of them from Llama. To choose sequences relevant to the study, we performed UMAP analysis of sequences for VHHs in the structure dataset using nanoBERT (24) embedding. Result (**Supplementary Figure S3A**) showed that VHHs from Dromedary and Bactrian clustered together as a separate cluster while there is no separation between sequences from Alpaca and Llama, which is not surprising as Alpaca and Llama are genetically close to each other (27). We filtered out sequences from Dromedary and Bactrian and generated a new dataset with 443 sequences for the study. Epitope and paratope residues were defined as all residues with an atom distance shorter than 4 Å between the antigen and antibody. CDRs were defined according to the IMGT numbering scheme.

To calculate distance between residues, central coordinate for each residue was obtained and distance between two central coordinates of two residues as the distance between two residues was calculated.

To calculate the buried surface area of the antigen (epitope) and VHH (paratope) in the complex, freeSASA (28) was used to calculate the solvent-accessible surface area (SASA) for antigen and VHH in the complex and as monomeric form. The buried surface area for antigen and VHH is the SASA of the monomeric form minus the corresponding SASA in the complex.

### 2.4 Molecular dynamics simulation

MD simulations were performed using Gromacs (29), 2024.1 version, following protocols described previously (30). Briefly, VHH atom coordinates for single chain were extracted from VHH crystal structure PDB files. VHH structure was placed in a cubic box with a water layer of 0.7 nm using OPLS-AA force field (31) and SPC water. Na+ Cl- ions were added to neutralize the system. The solvated, electroneutral system was energy minimized. NVT and NPT equilibrations were performed for 100 ps, followed by 100 ns production run at 300 K. The temperature was controlled with a modified Berendsen thermostat and the pressure with an isotropic Parrinello-Rahman at 1 bar.

### 2.5 Statistical tests

To assess significant correlation between groups, we calculated the Pearson’s correlation coefficient r and performed paired correlation test. To compare two groups of data, we mainly used two-tailed Mann-Whitney U test to assess significant difference, except those mentioned in the text. A P-value of less than 0.05 is considered to be significant. In figures, P-values are marked as followings: ns: P > 0.05; *: 0.01< P <=0.05; **: 0.001 <= P < 0.01; ***: 0.0001<= P < 0.001; ****: P <= 0.0001.

## 3 Results

### 3.1 Classical vs non-classical VHHs

Previous studies (9, 11, 32, 33) have reported the existence of non-classical VHHs, which lack VHH hallmark residues in FR2 region, in Camelid species such as Bactrian, Dromedary and Alpaca. To identify such sequences in our Alpaca VHH dataset, we used following criteria: a VHH sequence is considered as classical VHH if it has F/Y at position 42 (IMGT numbering), E/Q at position 49 and R at position 50, or if the best-matched germline gene is one of 17 VHH germlines (**Supplementary Table S3**) from Alpaca (18, 34); otherwise, the sequence will be considered as non-classical. We did not use the fourth hallmark residue (F/L/G in position 52) for identifying classical VHHs as the residue in that position is more various than other three based on previous study (12) and IGHV3S68*01, a VHH germline, has W instead of F/L/G in that position. With such criteria, 91.0% of sequences within dataset are classified as classical VHHs, which is consistent with published results (9, 11) where non-classical VHHs are considered as minority of the whole repertoire. Compared with classical VHHs, non-classical VHHs have significantly shorter CDR3 lengths and lower mismatch scores (**Table 1**). Similar results regarding CDR3 length have been reported previously (9). Lower mismatch scores indicated lower somatic mutations in non-classical VHHs. In addition, non-classical VHHs have significantly higher CDR3 net charge than classical VHHs (**Table 1**).

**Table 1:** Sequence feature differences between classical vs non-classical VHHs.

	Percentage	CDR3 length****	Number of mismatches****	CDR3 net charge****
Classical	91.0%	15.95 ± 0.01	11.00 ± 0.01	-0.654 ± 0.003
Non-classical	9.0%	13.93 ± 0.02	9.49 ± 0.01	-0.279 ± 0.007

Except percentage, other numbers are expressed as mean ± standard error, **** (P < 0.0001) indicating significantly different between two groups.

Top 5 germlines for non-classical VHHs are IGHV4S5*01 (19.5%), IGHV4S1*01(13.3%), IGHV3S39*01(8.9%), IGHV3S42*01 (7.7%) and IGHV3S1*01 (4.8%) (**Supplementary Table S4**). Similar to previous report (9), 11.2% of non-classical VHHs in our dataset have R instead of W at the first residue of FR4 region while only 2.7% of classical VHHs have such replacement. In Alpaca, there are no J genes with R at that position. To assess the possible mechanism for having R there, we analyzed codons at that location. 68.9% of sequences have CGG codon and 25.5% of sequences have AGG codon at that location, suggesting a single substitution of T->C or T-> A from TGG (W codon) as likely mechanism to have R there.

Classical VHHs used more restricted set of germlines and about 85% of them only used one of three germlines: IGHV3S53*01 (38.7%), IGHV3-3*01 (24.9%), IGHV3S65*01 (22.7%) (**Supplementary Table S4**), similar to previous reports in alpaca (11) and llama (10).

### 3.2 Distinct types of VHHs in alpaca

**Figure 1:**
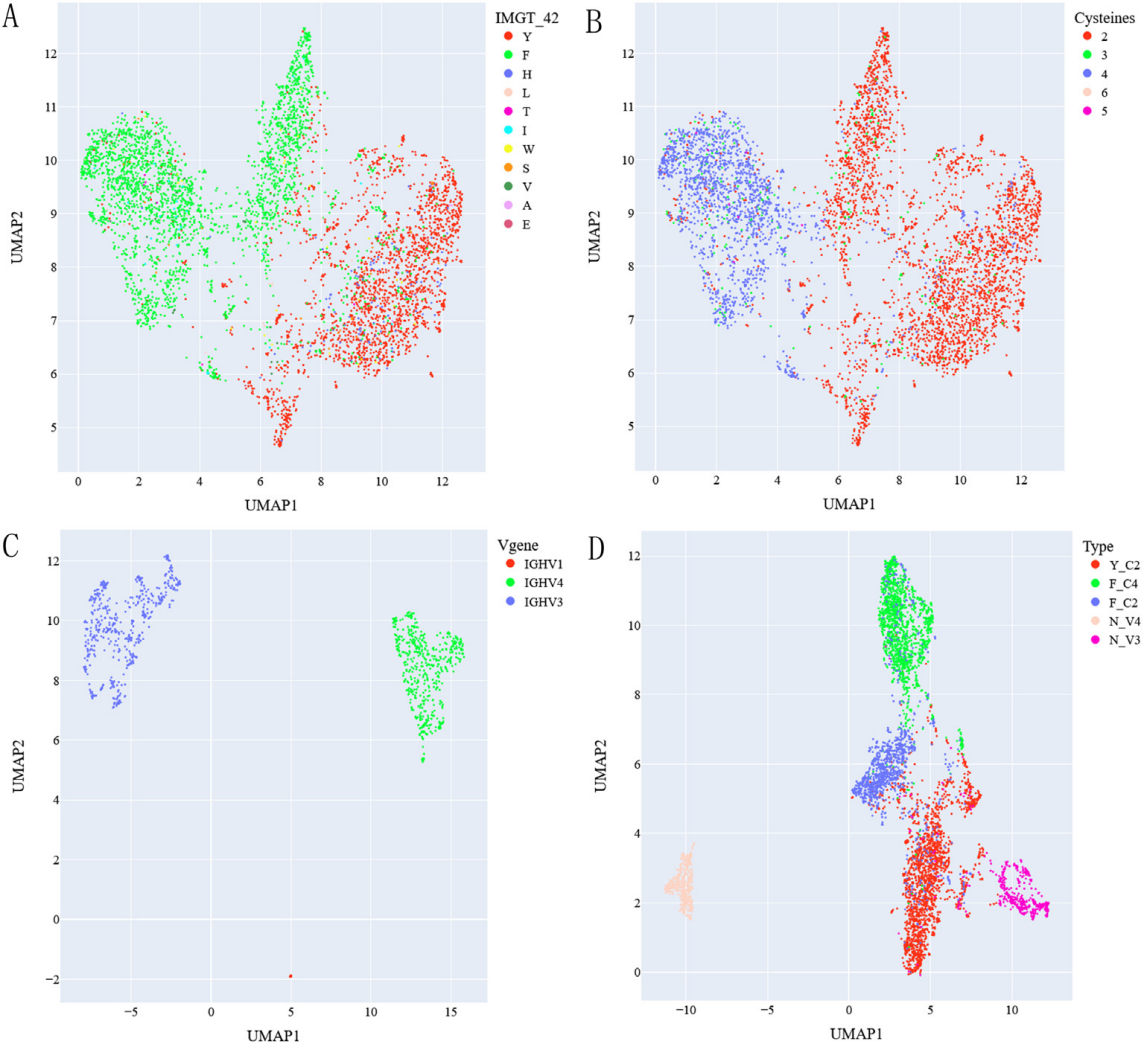
UMAP graph of top 5000 classical VHHs labeled with residue at IMGT 42 position **(A)** and number of cysteines **(B)** within sequences, top 1000 non-classical VHHs labeled with types of mapped V genes **(C)**. UMAP of classical and non-classical VHHs after removing non-assigned ones labeled with assigned types **(D)**. AntiBERTy is used to generate sequence embeddings.

We found that three germlines used by most classical VHHs can be identified by simple sequence features in FR2 region: residue (F or Y) at position 42 and number of cysteines in sequences. Correspondingly, three types of classical VHHs can be identified using such simple sequence features: Y_C2 VHHs with Y at position 42 and containing 2 cysteines in sequences; F_C2 VHHs with F at position 42 and containing 2 cysteines and F_C4 VHHs with F at position 42 and containing 4 cysteines. These three types of VHHs used IGHV3S53*01, IGHV3-3*01 and IGHV3S65*01 germlines respectively (**Supplementary Table S4**). To assess whether such classification is reasonable or not, we selected top 5,000 classical VHHs based on its frequency and visualized them in UMAP graph based on sequence embeddings generated by antibody/protein language models. **Figures 1A, B** showed UMAP graphs of these sequences using embeddings generated by AntiBERTy (23). The result showed three main clusters identifiable based on residues at position 42 (**Figure 1A**) and number of cysteines (**Figure 1B**) together. Similar results (**Supplementary Figures S1, S2**) were obtained using embeddings generated by nanoBERT (24) and ESM2 (25).

Applying the same UMAP analysis on top 1,000 non-classical VHHs, we found that they were clustered together based on type of V genes (IGHV1, 3, 4) they were mapped to (**Figure 1C**; **Supplementary Figures S1C, S2C**). As VHHs mapped to IGHV1 has less than 0.2% sequences in our dataset, we excluded them in our further analysis. So, for non-classical VHHs, we think there are two main types: N_V3-VHHs mapped to IGHV3 germlines; and N_V4-VHHs mapped to IGHV4 germlines. UMAP graph of 5 types of VHHs after excluding non-grouped ones (**Figure 1D**; **Supplementary Figures S1D, S2D**) showed 5 main clusters. N_V3 VHHs are closer to classical VHHs than N_V4, which is not surprising as all classical VHHs are based on IGHV3 germlines.

With such grouping criteria, 88.7% of VHHs (**Table 2**) in our dataset are grouped into one of 5 types. 98.6% of non-grouped ones are classical VHHs and 38.9% of them contain 3 cysteines, which cannot be grouped with current definition. Using age information of animals, we analyzed possible differences of group percentage in different age groups. No clear correlation was observed as animals age (**Table 2**), although Y_C2 VHHs tend to have lower percentage in 3-4 age group animals as compared to 0-1 and 1-2 age groups.

**Table 2:** Percentage of different VHH types in all animals and three age groups.

Type	All	Age 3-4	Age 1-2	Age 0-1
Y_C2	33.1%	30.1%	41.0%	38.7%
F_C2	22.7%	21.7%	35.6%	10.5%
F_C4	24%	27.2%	10.1%	26.0%
N_V3	5.5%	5.7%	3.2%	7.3%
N_V4	3.4%	3.0%	1.6%	8.0%

### 3.3 Sequence feature differences

**Figure 2:**
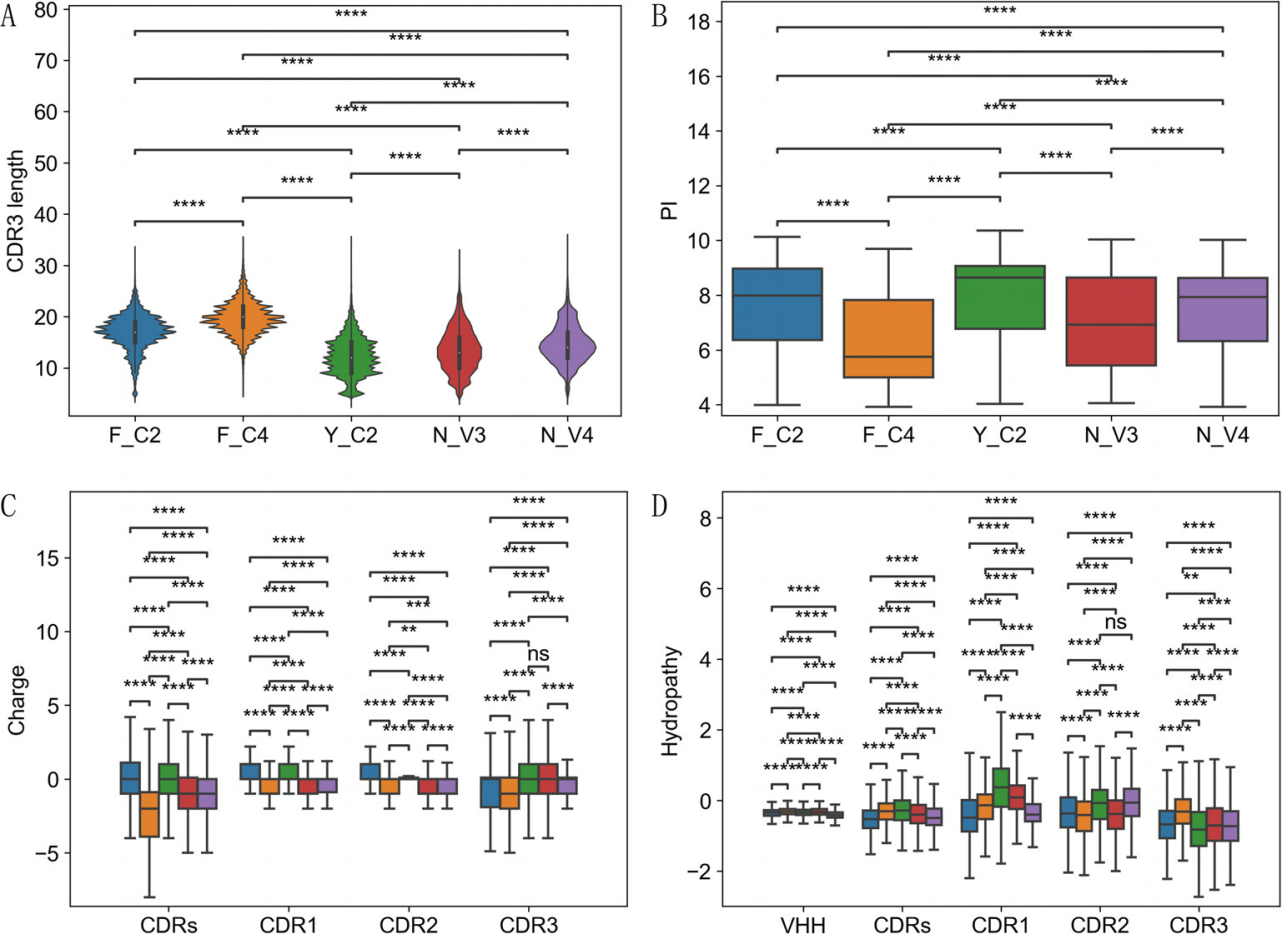
Sequence feature differences among 5 types of VHHs. Significant differences in all comparisons among 5 types were found for CDR3 length **(A)**, PI **(B)**. For charge **(C)**, significant differences in CDR1/2/3 and CDR regions among 5 types were found, except CDR3 charge in Y_C2 and N_V3 comparison. For hydropathy **(D)**, significant differences in CDR1/2/3/CDRs/VHH were found among 5 types, except CDR2 hydropathy in Y_C2 and N_V4 comparison. P-values are marked as followings: ns: P > 0.05; **: 0.001 <= P < 0.01; ***: 0.0001<= P < 0.001; ****: P <= 0.0001.

Most striking difference among these types of VHH is the CDR3 length (**Figure 2A**). F_C4 VHHs showed the longest CDR3 length than others, while Y_C2 VHHs showed the shortest CDR3 length, 6-7 residues shorter than F_C4 VHHs (**Figure 2A**). There are three clear distinct distributions of CDR3 length for Y_C2, F_C2 and F_C4 VHHs (**Figure 2A**). It is known that VHHs have longer CDR3 than conventional VH, and longer CDR3 is considered as one of means to compensate for the diversity loss due to lack of light chain (35). Such difference appears to be mainly contributed by F_C2 and F_C4 VHHs. In fact, Y_C2 VHHs on average have shorter CDR3 length than those in human or rabbit VH (36): 12.27 residues on average for Y_C2 VHHs vs 14.86 for Rabbit and 15.36 for Human. Two non-classical types of VHHs (N_V3 and N_V4) have an average CDR3 length between Y_C2 and F_C2 VHHs (**Figure 2A**) with significant differences (P < 0.0001) between them and with the other three types.

Another significant sequence feature difference among these types is the charge (**Figures 2B, C**). Y_C2 VHHs have the highest PI value among 5 types while F_C4 VHHs have the lowest (**Figure 2B**). The differences among 5 types are all significant (P < 0.0001). Consistent with PI value, we observed significant differences (P < 0.01) of charge among 5 types for all CDR regions (**Figure 2C**), except CDR3 region in Y_C2 and N_V3 comparison. F_C4 VHHs showed the lowest charge values in CDR, CDR1 and CDR3 (**Figure 2C**). Some of differences observed here can be easily explained by germlines these VHHs used. For example, F_C2 VHHs have the highest CDR1 net charge as the main germline (IGHV3-3*01) used contains an R residue, a positive charge residue, in CDR1 (**Supplementary Table S5**). F_C4 VHHs have the lowest CDR1 net charge as the main germline (IGHV3S65*01) used contains a D residue, a negative charge residue, in CDR1. Besides IGHV3S65, there are two more germlines (IGHV3S61 and IGHV3S66) used by F_C4 VHHs with significant percentages (**Supplementary Table S4**) and both contain negative charge residues in CDR1 (**Supplementary Table S5**). Lower PI value for F_C4 VHHs can be partially explained by lower net charge of germlines these VHHs use (**Supplementary Table S5**). However, the significant differences of CDR3 net charge among 5 types cannot be explained by germlines these VHHs used since CDR3 region is a result of VDJ recombination. There could be some selection pressure during repertoire development leading to such results.

We also observed significant differences in hydropathy indices among 5 types of VHHs (**Figure 2D**). Full length, CDRs and CDR1 hydropathy values displayed significant differences among all comparisons of 5 types of VHHs (P < 0.0001). For CDR2, except Y_C2 and N_V4 comparison, all other comparisons showed significant difference (P < 0.0001). For CDR3, all comparisons showed significant differences (P < 0.01). Among 3 classical types of VHHs, Y_C2 VHHs showed significantly higher hydropathy index (more hydrophobic) than other 2 types of VHHs in CDR1 and CDR2, while in CDR3, they showed significantly lower hydropathy index. F_C2 VHHs have the most hydrophilic CDRs and CDR1 sequences and F_C4 VHHs have most hydrophobic CDR3 sequences, but most hydrophilic CDR2 sequences. Since cysteine is a hydrophobic residue with a hydropathy index of 2.5 (22), and about 99% of F_C4 VHHs have cysteines in CDR3, it may not be very surprising to have high hydropathy value for CDR3 sequences of F_C4 VHHs. Similar to charge, some of differences among 5 types of VHHs observed here can be explained by the germlines these VHHs used. For example, the high hydropathy index in CDR1/CDR2 sequences of IGHV3S53 can explain why Y_C2 VHHs have a high hydropathy index in these two regions (**Supplementary Table S5**). However, not all observations can be explained by the differences in the germline used, especially the significant differences observed for CDR3 hydropathy indices among 5 types of VHHs. Similar to CDR3 charge differences, selection pressure during repertoire development may shape different types of VHHs differently.

### 3.4 Hinge usage and somatic mutation differences

Two types of hinges (2B - hinge for IgG2b isotype, and 2C - hinge for IgG2c isotype, **Supplementary Table S6**) are used in Alpaca VHHs (34). All sequences in the dataset we analyzed in this study contain either 2B or 2C. Overall, 56% sequences have 2B hinge and 44% sequences 2C hinge. For 5 types of VHHs, there appears to have some preference. Two non-classical types of VHHs are more likely to have 2C hinge than three classical types of VHHs based on paired T test of 2C hinge percentage of each type of VHHs among 22 animals (**Table 3**, P < 0.002)). Among three types of classical VHHs, F_C4 VHHs are more likely to have 2C hinge than the other 2 types of VHHs (P < 0.05, paired T-test). It is not clear the biological significance of such results.

**Table 3:** Average hinge usage in 5 types of VHHs among 22 animals.

	Y_C2	F_C2	F_C4	N_V3	N_V4
2B	59.3%	57.4%	46.4%	31.3%	28.4%
2C	40.7%	42.6%	53.6%	68.7%	63.6%

Mismatch score, number of mismatches over 100 bp alignment with germline, measures somatic hypermutation (SHM) within sequences. **Table 4** summarizes mismatch score differences among 5 types of VHHs and corresponding results in three age groups. As expected, when animals age, repertoire accumulates more somatic mutations, and we observed a clear increase in mismatch scores among all 5 types of VHHs as animals age. Three types of classical VHHs have significantly higher mismatch scores than non-classical ones (P < 0.0001), consistent with **Table 1** result. Among 3 types of classical VHHs, Y_C2 VHHs have the highest mismatch scores in all animals and 2 out of 3 age groups. Possibly because of small number of animals in Age 1-2 and 0-1 groups, there are some variations observed, not consistent with overall results.

**Table 4:** Mismatch score differences among 5 types of VHHs and corresponding results in three age and two hinge groups.

Type	All ****	Age 3-4	Age 1-2	Age 0-1	2B	2C****
Y_C2	11.31 ± 0.01	13.25 ± 0.02	8.60 ± 0.02	6.82 ± 0.02	9.78 ± 0.02	14.19 ± 0.02
F_C2	10.85 ± 0.02	12.23 ± 0.02	8.45 ± 0.02	6.20 ± 0.03	9.60 ± 0.02	13.28 ± 0.02
F_C4	10.24 ± 0.01	11.14 ± 0.01	8.73 ± 0.04	5.80 ± 0.02	9.06 ± 0.02	11.27 ± 0.02
N_V3	9.95 ± 0.03	11.26 ± 0.04	7.49 ± 0.07	6.07 ± 0.04	9.79 ± 0.06	10.02 ± 0.04
N_V4	8.99 ± 0.04	10.61 ± 0.05	7.19 ± 0.10	5.65 ± 0.04	8.58 ± 0.07	9.22 ± 0.04

Numbers are expressed as mean ± standard error, **** (P < 0.0001) indicating significantly different among all group comparisons.

Henry et al. (10) and some conference presentations have shown that VHHs with 2C hinge have significantly higher somatic mutation than VHHs with 2B hinge in Llama. We observed similar results in Alpaca: mean mismatch score for 2B hinge is 9.66, significantly lower than mean mismatch score for VHHs with 2C hinge which is 12.40. Such mismatch score differences between two hinge types were also observed for 5 types of VHHs (**Table 4**). Among all types of VHHs, Y_C2 VHHs with 2C hinge have the highest mismatch scores.

### 3.5 CDR3 conformation differences

Previous study (37) suggested that VHH CDR3 may adopt concave, loop or convex structure configurations. More recently, Dizicheh et al. (16) analyzed VHH CDR3 conformation and found two main CDR3 conformations: extended and kinked. To study possible structure feature differences among 5 types of VHHs as well as possible correlations with previous studies, we used VHH structure data from SAbDab-nano (26, 38) for the analysis. Using the same criteria as those used in NGS data analysis, we assigned different types to these sequences. 406 sequences were assigned to 4 types of VHHs (**Supplementary Table S7**) and such assignment was confirmed by UMAP analysis (**Supplementary Figure S3B**). The full list of structural dataset is available in **Supplementary Table S8**. We did not find any N_V4 sequences in the structure dataset, thus this type of VHH was excluded in following structure analyses. Same main sequence feature differences including CDR3 length, charge, hydropathy (**Supplementary Figure S4**) among different types of VHHs were found in this small dataset although differences were not as significant as those in NGS dataset.

**Figure 3:**
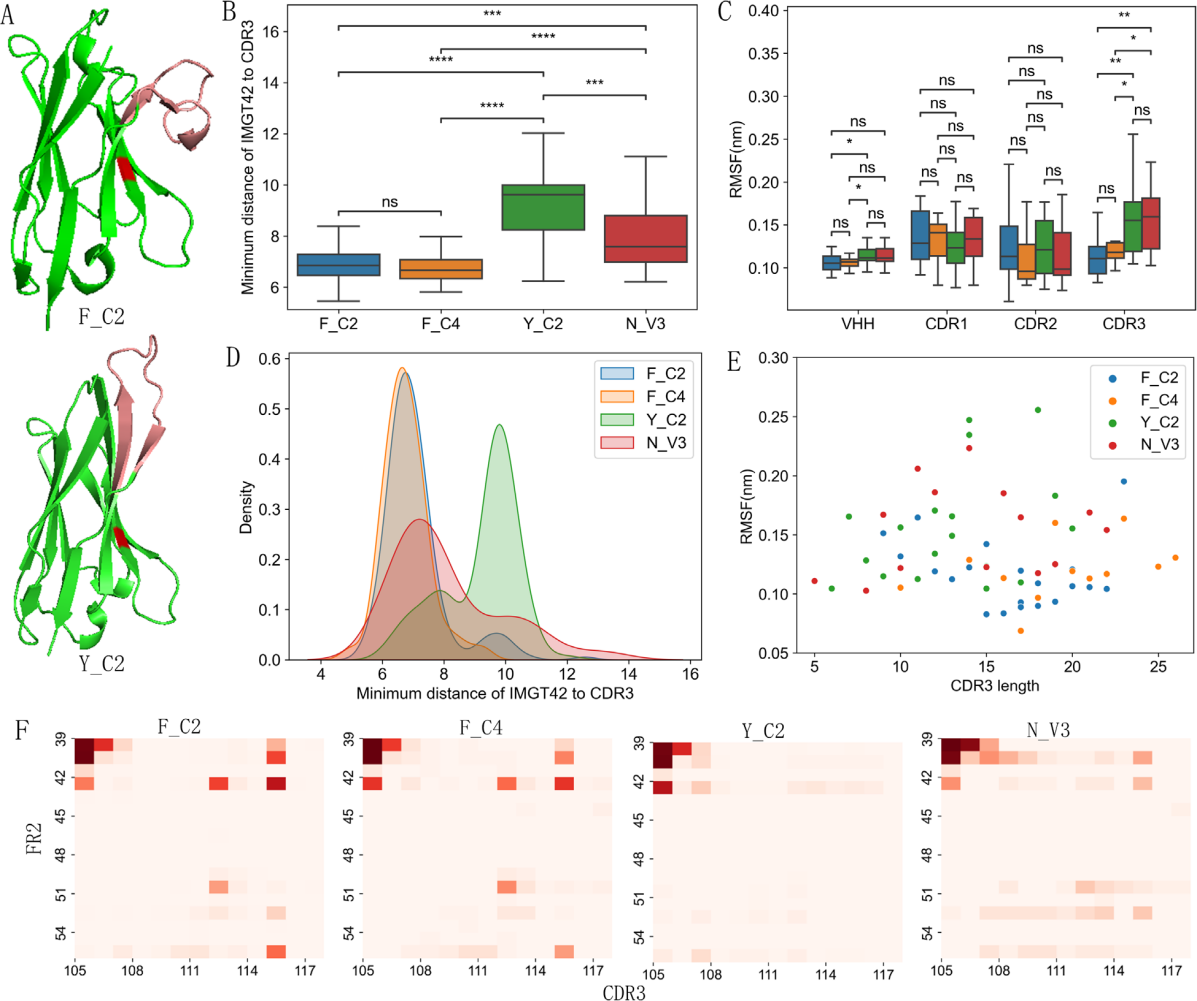
Structure feature differences among 4 types of VHHs. **(A)** Examples of VHHs with bent down (PDB ID: 6HJY) and extended (PDB ID: 6GKD) CDR3 (pink colored) conformation. Residue at IMGT 42 position is red colored. Y_C2 VHHs showed largest minimum distance between residue at 42 position and CDR3 among 4 types of VHHs based on both boxplot **(B)** and density map **(D)**. Y_C2 and N_V3 showed larger flexibility in CDR3 as compared to other two types **(C)**. No significant correlation was observed between flexibility and CDR3 length **(E)**. Heat map showing the probability of interaction between FR2 and CDR3 residues **(F)**. F_C2 and F_C4 VHHs showed more interactions between FR2 and CDR3 residues as compared to Y_C2 and N_V3. P-values are marked as followings: ns: P > 0.05; *: 0.01< P <=0.05; **: 0.001 <= P < 0.01; ***: 0.0001<= P < 0.001; ****: P <= 0.0001.

While inspecting example VHH structures visually, we noticed that Y_C2 VHHs are more likely to have CDR3 extended away from FR2 (**Figure 3A**) while F_C2 VHHs are more likely to have CDR3 bending down to cover FR2 area (**Figure 3A**). To quantitatively measure such differences, we calculated the minimum distance between residue at IMGT 42 and any residue in CDR3 after excluding the first and last 2 residues of CDR3. Y_C2 VHHs clearly showed significantly larger distance as compared to other 3 types of VHHs (**Figures 3B, D**), suggesting that most of Y_C2 VHHs have CDR3 extended away from FR2. F_C2 and F_C4 have smaller distances and narrow peak in density distribution, suggesting that most of these VHHs will have CDR3 bent down toward FR2 (**Figures 3B, D**). The values for N_V3 VHHs are between the above two groups. When CDR3 bent down toward FR2, we expect some interactions between residues of these two fragments. With 4Å as distance cutoff, we analyzed possible molecular interactions between residues in FR2 and CDR3 among 4 types of VHHs. Indeed, we observed many possible interactions between FR2 (IMGT 39, 40, 42, 50, 55) and CDR3 (IMGT 112, 115) residues in F_C2 and F_C4 VHHs (**Figure 3F**). Such interactions are minimal or non-existent in Y_C2 VHHs (**Figure 3F**). Structurally F and Y are similar (**supplementary Figure S5**), hydroxylation of F becomes Y. Y is amphipathic while F is hydrophobic, which is probably one main reason why CDR3 bent down to cover the residue in F_C2 and F_C4 VHHs.

Different CDR3 structure may indicate different CDR3 flexibility, which may impact conformational stability, binding affinity, kinetic stability etc. (39, 40). To assess such possibility, we selected 3D structures of several VHHs from dataset that includes each length of CDR3 of 4 types of VHHs. 100 ns MD simulations were performed on these VHH structures and root mean square fluctuations (RMSF) of whole VHH and CDR regions were used to assess the flexibility of these regions (**Figures 3C, E**). CDR3 of Y_C2 and N_V3 VHHs showed significantly higher RMSF than CDR3s in F_C2 and F_C4 VHHs (**Figure 3C**). No significant differences were observed for CDR1 and CDR2. Such results suggest that Y_C2 and N_V3 VHHs have more flexible CDR3, consistent with the result that their CDR3s are more likely to be in extended conformation. There is no significant correlation observed between CDR3 length and RMSF value for each individual type or whole set (**Figure 3E**).

### 3.6 VHH-antigen interface differences

**Figure 4:**
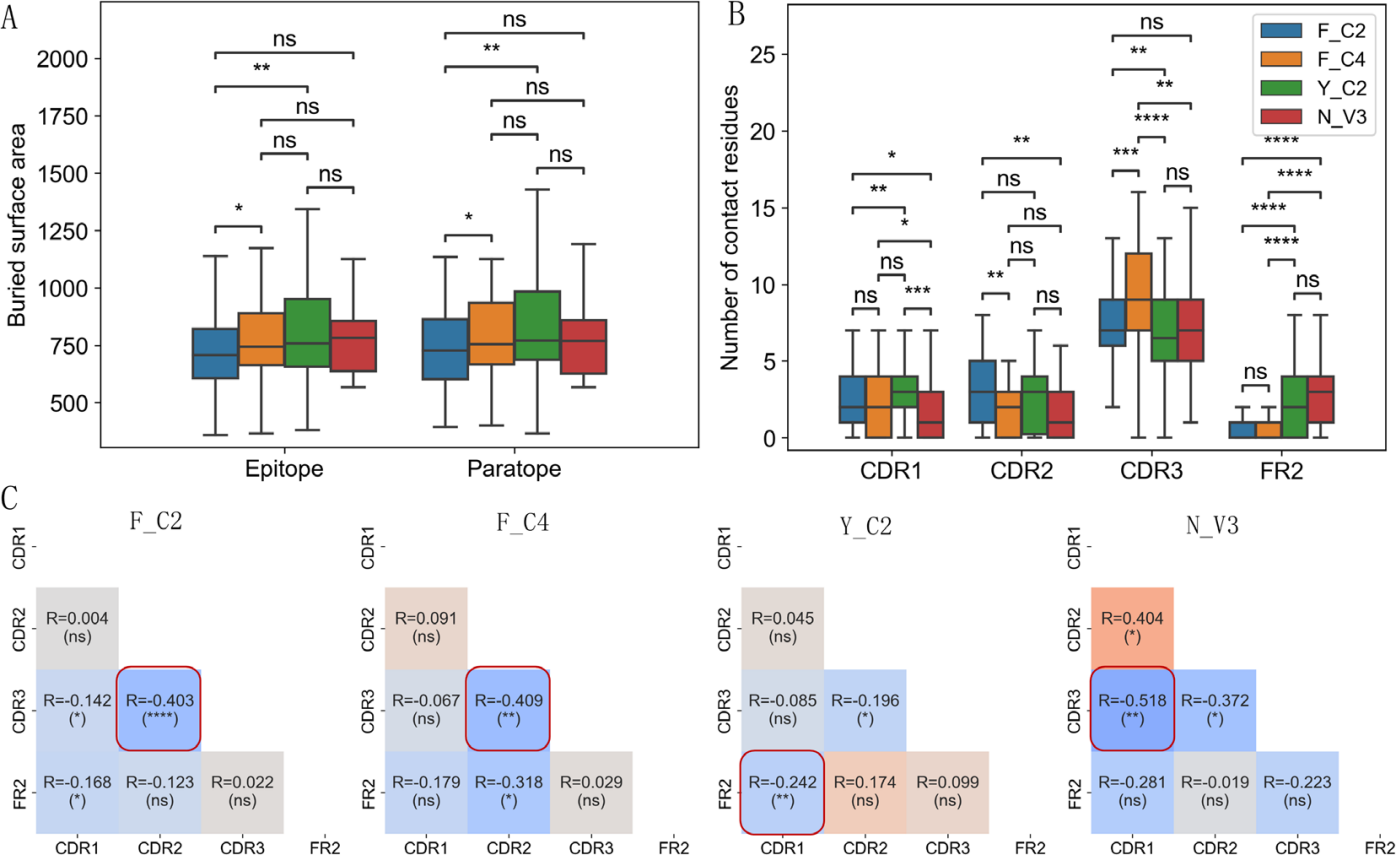
Interaction interface differences among 4 types of VHHs. Y_C2 VHHs showed slightly larger epitope and paratope size as measured by buried surface area **(A)**. Number of contact residues in CDR1/2/3 and FR2 regions showed significant differences among 4 types of VHHs **(B)**. The correlations of number of contact residues among 4 regions (CDR1, CDR2, CDR3, FR2) **(C)** showed distinct dependency of contact residues among 4 types of VHHs. Most significant correlation for each type is highlighted with red box. P-values are marked as followings: ns: P > 0.05; *: 0.01< P <=0.05; **: 0.001 <= P < 0.01; ***: 0.0001<= P < 0.001; ****: P <= 0.0001.

Using VHH-antigen complex 3D structures in our dataset, we analyzed possible differences in interface characteristics among 4 types of VHHs. Using either the number of contact residues (**Supplementary Figure S6A**) or buried surface area in the interface (**Figure 4A**), we compared the interface size among 4 types of VHHs. Y_C2 VHHs consistently showed slightly larger paratopes than other types, and significantly larger epitope and paratope sizes than those of F_C2 VHHs (**Figure 4A**). Such result is unexpected as Y_C2 VHHs have the shortest CDR3 length among all types of VHHs. Detailed location analysis of contact residues displayed more distinct differences among 4 types of VHHs (**Figures 4B, C**). As expected, CDR3 region contributed most to the interaction among the 4 regions we analyzed (**Figure 4B**). F_C4 VHHs have the highest number of contact residues in CDR3 while Y_C2 have the lowest. Such results are not surprising as F_C4 VHHs have the longest CDR3 length while Y_C2 VHHs have the shortest CDR3 length. Number of contact residues in CDR3 showed significant correlation with its length (**Supplementary Figure S7**). Y_C2 and N_V3 VHHs also have significantly higher number of contact residues in FR2 as compared to other 2 types. F_C2 VHHs use significantly more residues in CDR2 as compared to F_C4 and N_V3 VHHs, and Y_C2 VHHs use significantly more residues in CDR1 as compared to F_C2 and N_V3 VHHs.

Further correlation analysis among number of contact residues in CDRs and FR2 regions showed additional patterns (**Figure 4C**). A negative correlation was observed between the number of contact residues in CDR2 and CDR3 for all types of VHHs with F_C2 showing the most significant negative correlation. The most significant negative correlation for Y_C2 VHHs is between CDR1 and FR2, and for N_V3 VHHs is between CDR1 and CDR3. Overall, such results suggested that residues in CDR2/CDR3 of VHHs work in a complemental manner when contributing residues to the antigen binding. Different types of VHHs have their own uniqueness in the region to use when contributing residues to the antigen binding.

### 3.7 Two types of binding paratopes and their correlation with different types of VHHs

**Figure 5:**
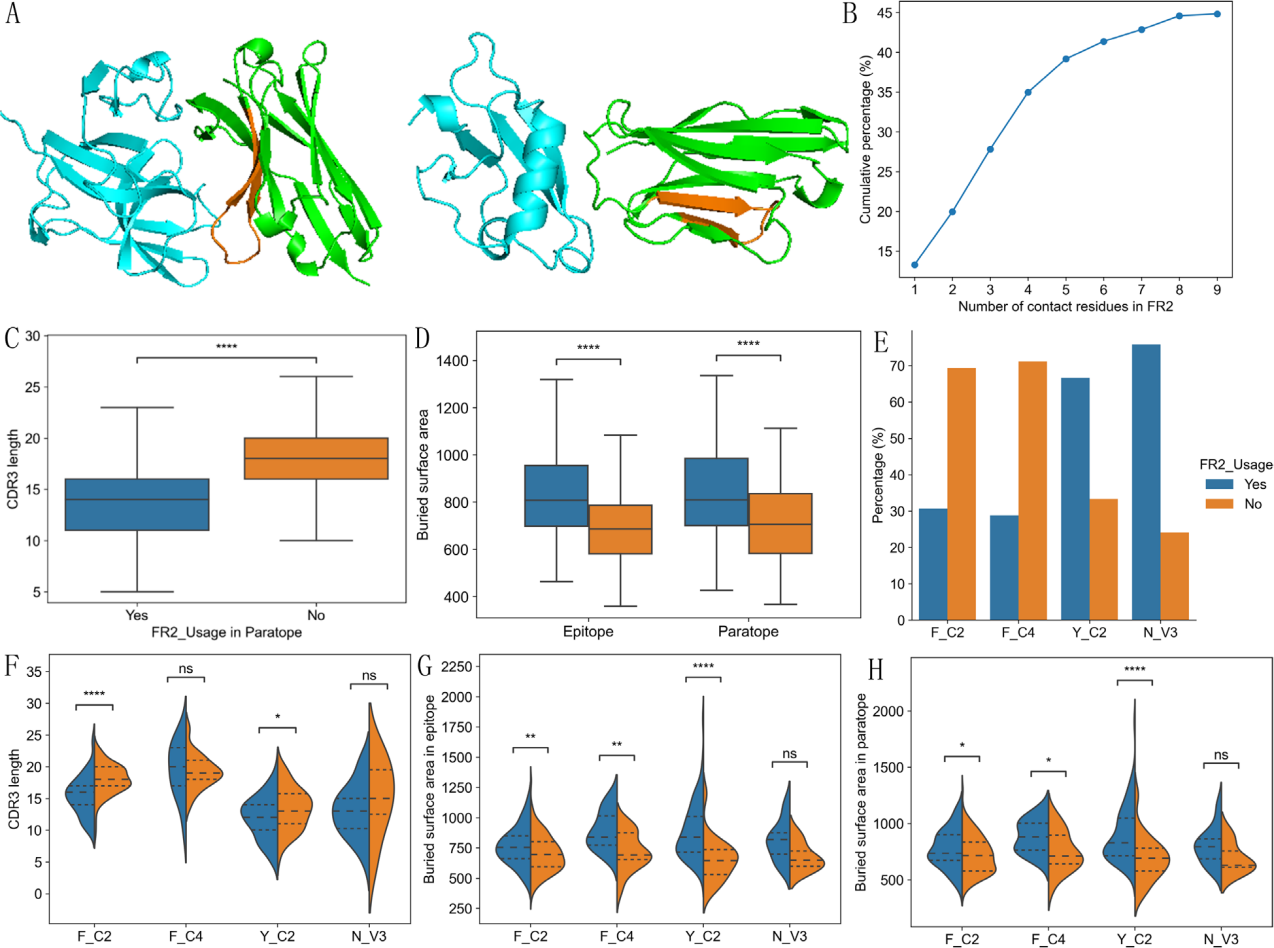
Two types of binding paratopes and their differences among different types of VHHs. Example structures with contact residues from FR2 (PDB ID: 7B5G, FR2 region orange colored) vs not (PDB ID: 5IP4, FR2 region orange colored) **(A)**. Cumulative percentage of structures with different number of contact residues from FR2 **(B)**. VHHs with contact residues in FR2 have significantly shorter CDR3 **(C)** and larger epitope and paratope sizes **(D)**. Y_C2 and N_V3 are more likely to have FR2 contact residues than F_C2 and F_C4 VHHs **(E)**. F_C2 and Y_C2 VHHs using FR2 in binding have shorter CDR3 **(F)**. VHHs using FR2 in binding have larger epitope **(G)** and paratope **(H)** sizes. P-values are marked as followings: ns: P > 0.05; *: 0.01< P <=0.05; **: 0.001 <= P < 0.01; ****: P <= 0.0001.

FR2 involvement in antigen binding is unique feature of VHHs as FR2 in conventional antibodies is covered by light chain and thus is not involved in antigen binding. Based on such results, we think VHH paratopes can be grouped into two kinds, one involving FR2 (**Figure 5A**), and the other not (**Figure 5A**). 45% of VHHs in structure dataset involve FR2 in antigen binding with 1 to 9 contact residues (**Figure 5B**). They have significantly shorter CDR3 (**Figure 5C**, P< 0.0001), and larger binding interfaces (**Figure 5D**; **Supplementary Figure S6B**, P < 0.0001). Such results suggest that VHHs with short CDR3 may have large binding interface by using residues in FR2, which explains the observation that Y_C2 VHHs have large binding interface. VHHs not using FR2 residues in binding tend to use CDR1 residues more (**Supplementary Figure S8A**).

Consistent with contact residue analysis, Y_C2 and N_V3 VHHs are more likely to involve FR2 in binding as compared to F_C2 and F_C4 VHHs (**Figure 5E**, P < 0.0001). 2 out of 4 types of VHHs showed significantly shorter CDR3 when FR2 is involved in binding (**Figure 5F**). Significant larger binding interfaces were observed in 3 out of 4 types of VHHs when using FR2 in binding (**Figures 5G, H**).

**Figure 6:**
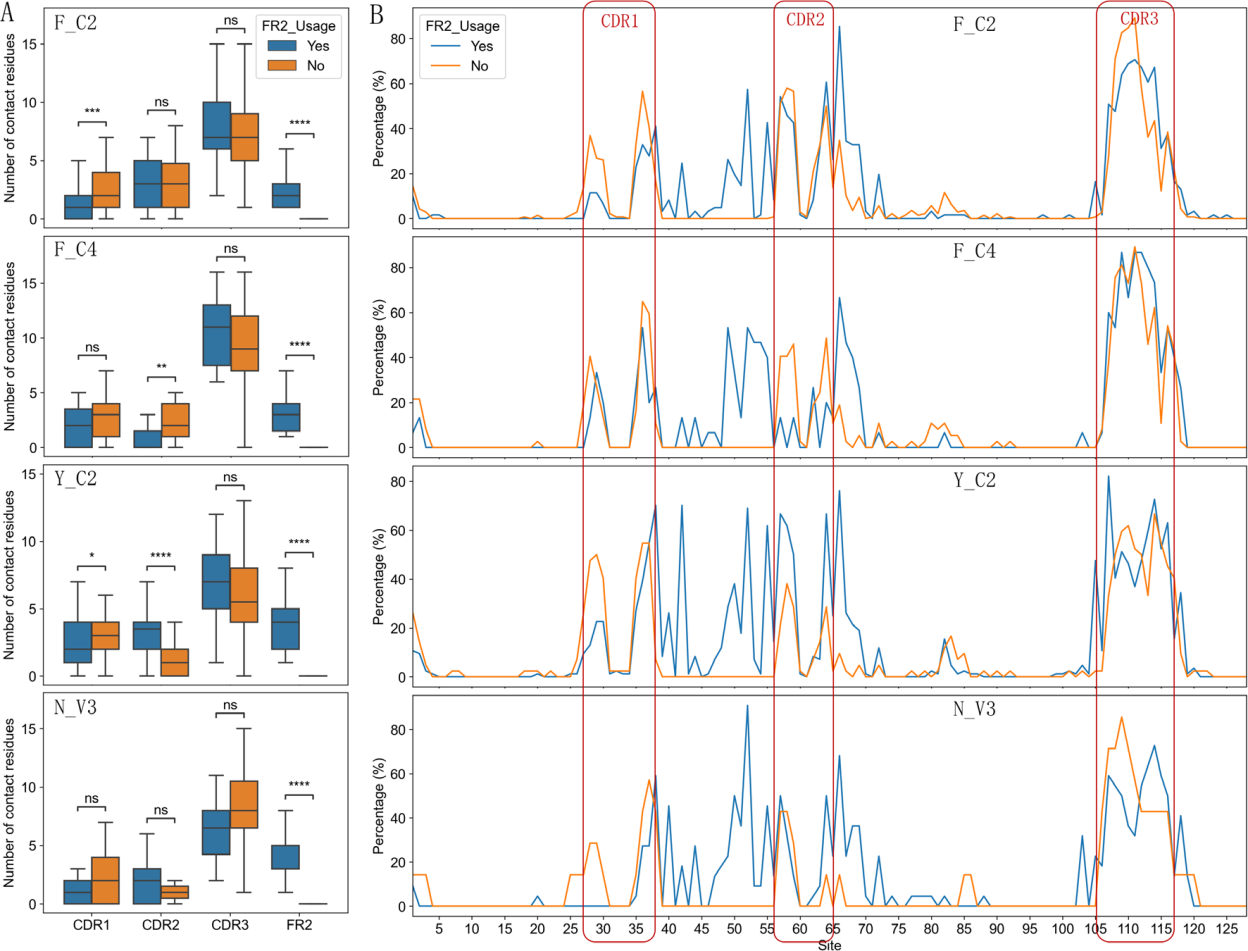
Comparison of number of contact residues in CDR1/2/3 and FR2 regions **(A)** and contact residue distribution **(B)** between two types of paratopes for each type of VHHs. The Y-axis **(B)** represents the percentage of VHHs involved in binding at a specified position. P-values are marked as followings: ns: P > 0.05; *: 0.01< P <=0.05; **: 0.001 <= P < 0.01; ***: 0.0001<= P < 0.001; ****: P <= 0.0001.

Although VHHs not using FR2 residues in binding tend to use CDR1 residues more (**Supplementary Figure S8A**), different types of VHHs showed different preferences in contributing residues to the binding among two types of paratopes (**Figure 6A**). F_C4 VHHs used CDR2 residues significantly more in VHHs not using FR2 residues in binding. Surprisingly, Y_C2 VHHs used CDR2 residues significantly less (P < 0.0001) in VHHs not using FR2 residues in binding, suggesting that Y_C2 VHHs not using FR2 residues in binding mainly used residues from CDR1 and CDR3 in binding and such VHHs are expected to have smaller paratopes. Indeed, Y_C2 VHHs showed the most significant differences in interface size between two types of paratopes (**Figures 5G, H**) among 4 types of VHHs.

More detailed analysis of contact residue distribution within full length VHHs showed additional distinct patterns (**Figure 6B**; **Supplementary Figure S8B**). Overall, residues in IMGT positions 52, 55 and 42 in FR2 contributed most to the antigen binding in VHHs which use FR2 for the binding (**Supplementary Figure S8B**). It appears that VHHs without using FR2 in binding have similar contact residue distribution among 4 types of VHHs and are similar to VH of conventional antibodies (12, 14). VHHs using FR2 in binding have their own uniqueness. Y_C2 VHHs mostly use residue at positions 42, 52 and 55 while the other three types VHHs do not use 42 as one of top three residues (**Table 5**). The residue at 42 position in Y_C2 VHHs is Y. Such result suggests that this Y residue is involved in binding in 47% of all Y_C2 VHHs, a very significant contribution to the binding from single residue. N_V3 VHHs use residue at position 40 quite significantly while it is not the case for other types. F_C4 VHHs use residue at positions 49 and 53 quite significantly while it is not the case for other types.

**Table 5:** Top 3 FR2 residues used in binding among 4 types of VHHs using FR2 in binding.

		F_C2	F_C4	Y_C2	N_V3
First	IMGT Position	52	52	42	52
	Percentage	57%	50%	70%	91%
Second	IMGT Position	55	49	52	50
	Percentage	43%	50%	69%	50%
Third	IMGT Position	49	53	55	40/55
	Percentage	26%	43%	62%	45%

## 4 Discussion

In this study, we found that VHHs in Alpaca can be grouped into 5 types, 3 types for classical and 2 types for non-classical VHHs, with distinct sequence and structure features. The three classical types of VHHs can be identified easily using simple sequence features: residue at IMGT position 42 and number of cysteines. These 5 types showed significant differences in CDR3 length, net charges, and hydrophobic properties. Although some of the differences can be explained by germlines they used, CDR3-related properties (length, net charge and hydropathy index) cannot be explained by germlines as CDR3 is the result of VDJ recombination. VHHs are known to have long CDR3, however, Y_C2 VHHs have very short CDR3, shorter than those in human and rabbit VHs. Two main conformations of CDR3 are found in VHHs: extended away from FR2 and bent down toward FR2, similar to “extended and kinked” conformations reported by Dizicheh et al. (16). We further showed that Y_C2 VHHs are more likely to have extended CDR3 conformation and such conformation is more flexible.

One unique feature in VHH paratope is the involvement of FR2 residues in antigen binding for some VHHs. Such paratope is not possible in conventional antibodies as FR2 is involved in interacting with light chain. Y_C2 and N_V3 VHHs are more likely (>65%) to have FR2 residues in their binding interface than F_C2 and F_C4 VHHs (<30%). More interestingly, binding interfaces containing FR2 residues are significantly larger than those without, which explains the unexpected observation that VHHs with short CDR3 lengths have larger binding interfaces. In other words, one novel binding paratope that only existed in VHHs not in conventional antibodies is mainly contributed by VHHs with short CDR3. Because of lacking light chain and more restricted germline usage, it is expected that VHH sequence diversity may be lower than conventional antibodies. Using FR2 residues extensively in antigen binding suggests that VHHs will have higher paratope diversities. Indeed, study (14) showed greater diversity of nanobody paratopes as compared to conventional antibodies.

Among 5 types of VHHs described in this study, Y_C2 is probably the most unique one. As one of three types of classical VHHs, it has a third of sequences (33.1%) in repertoire. However, with its short CDR3 length, it is against general understanding of a single-domain antibody, which is known for its long CDR3. It uses more CDR1/FR2 residues in antigen binding than other types of classical VHHs, possibly to compensate for the short CDR3 length. Extended CDR3 together with FR2 may form a large concave shaped paratope (ex. 7YAG) (**Supplementary Figure S9A**). Short CDR3 together with CDR1 and CDR2 may form a flat (ex. 7KJI) (**Supplementary Figure S9B**), or convex (ex. 7RNN) (**Supplementary Figure S9C**) shaped paratopes. Extended CDR3 conformation with medium length of CDR3 may form a loop sticking deep into cavities of an antigen (ex. 6RTY) (**Supplementary Figure S9D**). The paratopes of Y_C2 VHHs appear to be very versatile with distinct shapes. All paratope shapes suggested in the study (37) can be found in Y_C2 VHHs. In contrast, for F_C2 and F_C4 VHHs with CDR3 bend down to cover FR2, the main paratope shapes may be convex shaped (ex. 7B2Q) (**Supplementary Figure S9E**).

In this study, we focused on VHHs from Alpaca. Similar results are expected for VHHs in Llama based on internal analysis of unpublished sequence data as well as structure data analyzed in this study. VHHs from Bactrian and Dromedary camels form their own clusters in UMAP analysis, suggesting these VHHs may have their own unique sequence and structure features, which warrants a separate study. Indeed, from published germline sequences for Bactrian (41) and Dromedary (18), we found all germlines coding for classical VHHs contain 3 or more cysteines. Such results suggest that Y_C2 and F_C2 types of VHHs analyzed here will not be major populations in Camels.

Novel findings in this study will enhance our understanding of VHHs in Alpaca and guide us during VHH discovery, engineering, and design processes. For example, during discovery, we may want to select specific types of VHHs based on the shape of epitopes to increase the chance of success. During hits optimization to reduce potential immunogenicity and improve developability, we want to avoid changing residues in FR2 for Y_C2 VHHs, especially those residues with a high probability of interacting with antigens. During VHH designs, we may choose a specific combination of template and CDR3 length based on the shape of targeted epitopes. We have implemented some of above findings in our VHH discovery and development platform to more efficiently engineer VHHs in large scale.

## 5 Conclusions

In this study, we systematically analyzed different types of VHHs in Alpaca, identifiable using simple sequence features and showed distinct sequence and structure feature differences among them. Furthermore, we compared two kinds of paratopes in VHHs and their usage in different types of VHHs. We found that paratopes involving FR2 residues are used mostly by VHHs with short CDR3. This type of VHH may enable us to design novel therapies with distinct binding modalities.

## Data Availability

Raw sequencing data generated from this study were submitted to Sequence Read Archive (SRA) under accession number PRJNA1148326.
