# Supplementary material for: Distinct types of VHHs in Alpaca
Source: Front Immunol. 2024 Nov 12;15:1447212. doi: 10.3389/fimmu.2024.1447212 (PMC11588638; doi:10.3389/fimmu.2024.1447212)
Supplement: Supplementary file 1 [file Presentation1.pptx]

## Slide 1
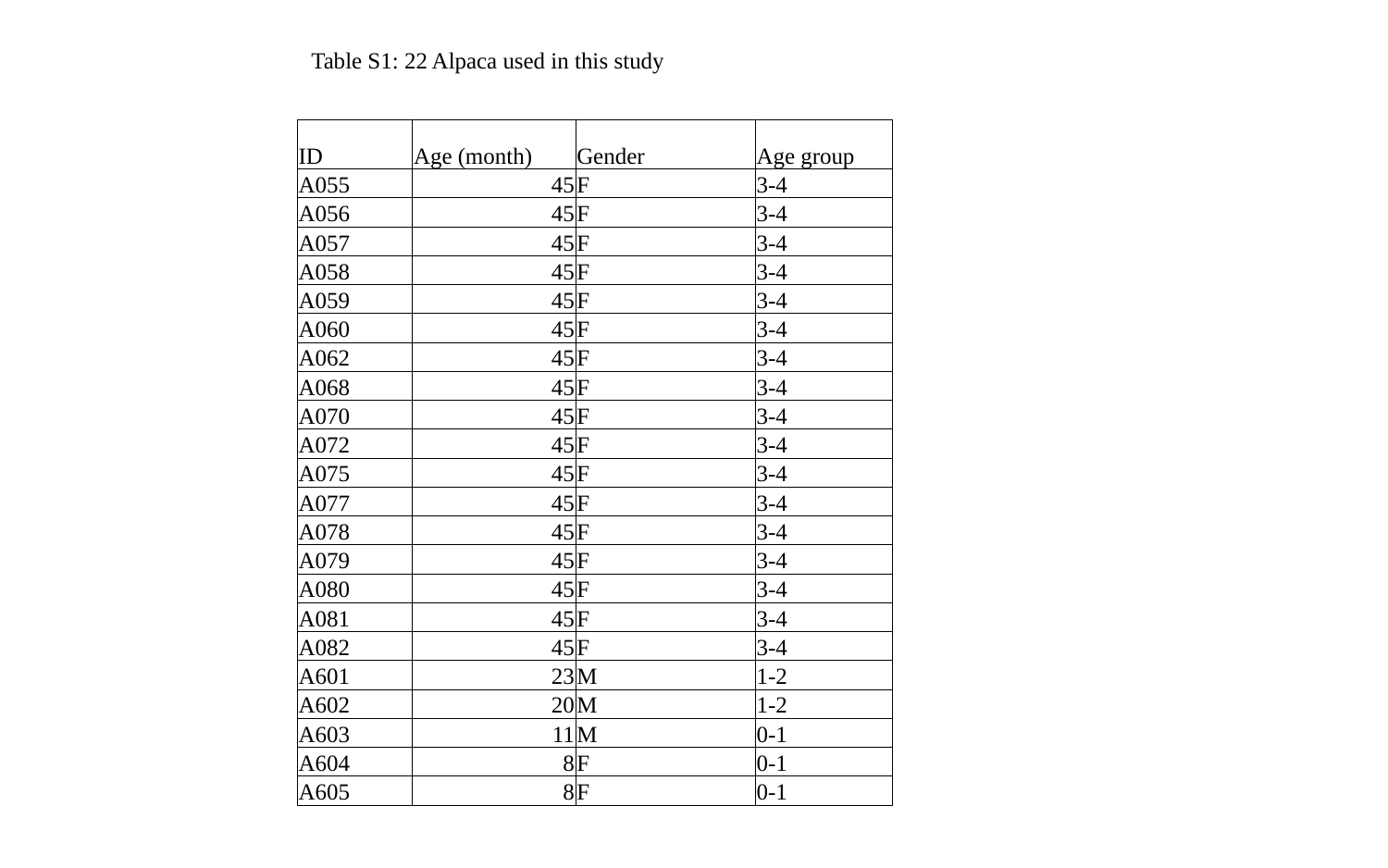

Table S1: 22 Alpaca used in this study
| ID | Age (month) | Gender | Age group |
| --- | --- | --- | --- |
| A055 | 45 | F | 3-4 |
| A056 | 45 | F | 3-4 |
| A057 | 45 | F | 3-4 |
| A058 | 45 | F | 3-4 |
| A059 | 45 | F | 3-4 |
| A060 | 45 | F | 3-4 |
| A062 | 45 | F | 3-4 |
| A068 | 45 | F | 3-4 |
| A070 | 45 | F | 3-4 |
| A072 | 45 | F | 3-4 |
| A075 | 45 | F | 3-4 |
| A077 | 45 | F | 3-4 |
| A078 | 45 | F | 3-4 |
| A079 | 45 | F | 3-4 |
| A080 | 45 | F | 3-4 |
| A081 | 45 | F | 3-4 |
| A082 | 45 | F | 3-4 |
| A601 | 23 | M | 1-2 |
| A602 | 20 | M | 1-2 |
| A603 | 11 | M | 0-1 |
| A604 | 8 | F | 0-1 |
| A605 | 8 | F | 0-1 |

## Slide 2
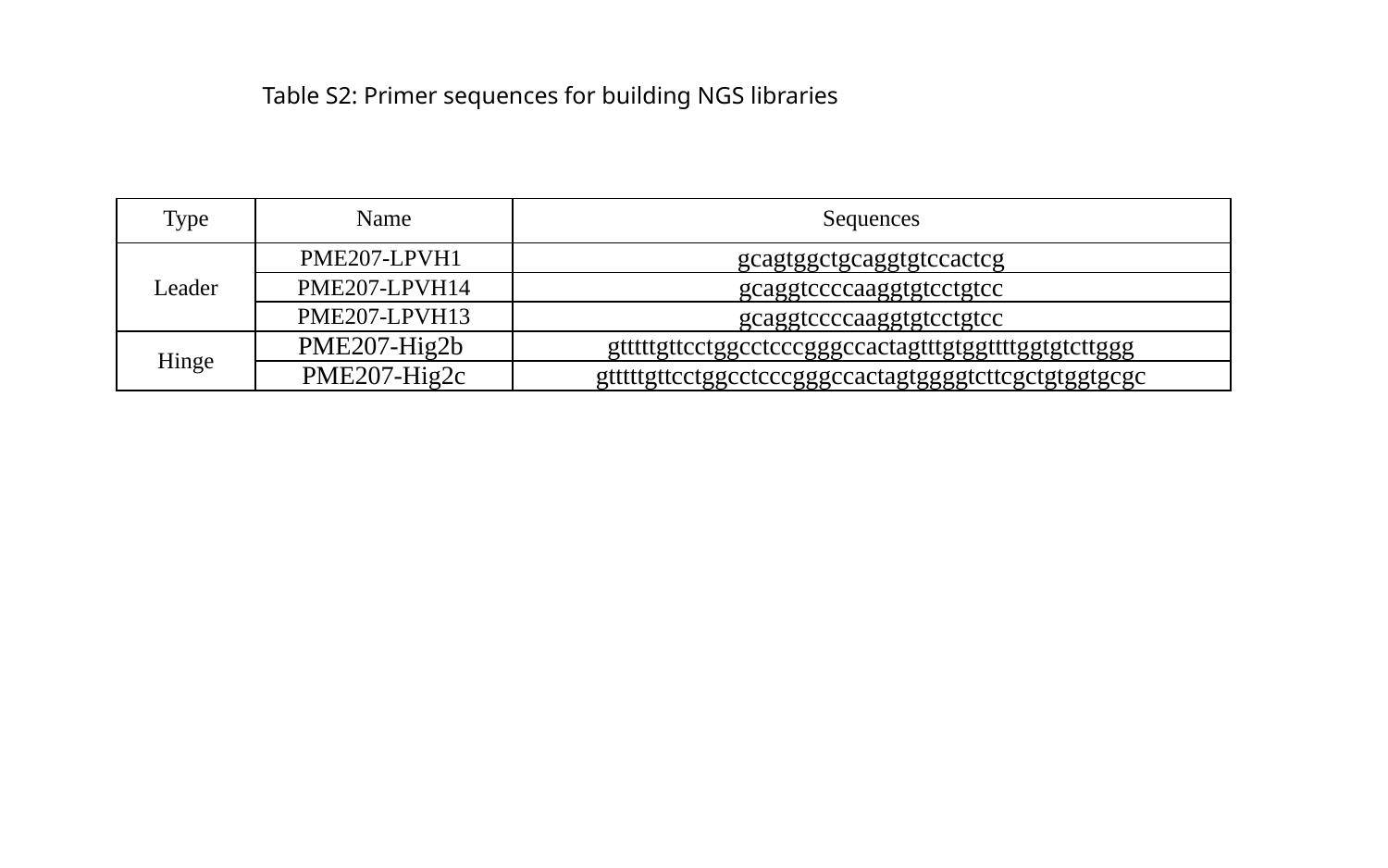

Table S2: Primer sequences for building NGS libraries
| Type | Name | Sequences |
| --- | --- | --- |
| Leader | PME207-LPVH1 | gcagtggctgcaggtgtccactcg |
| | PME207-LPVH14 | gcaggtccccaaggtgtcctgtcc |
| | PME207-LPVH13 | gcaggtccccaaggtgtcctgtcc |
| Hinge | PME207-Hig2b | gtttttgttcctggcctcccgggccactagtttgtggttttggtgtcttggg |
| | PME207-Hig2c | gtttttgttcctggcctcccgggccactagtggggtcttcgctgtggtgcgc |

## Slide 3
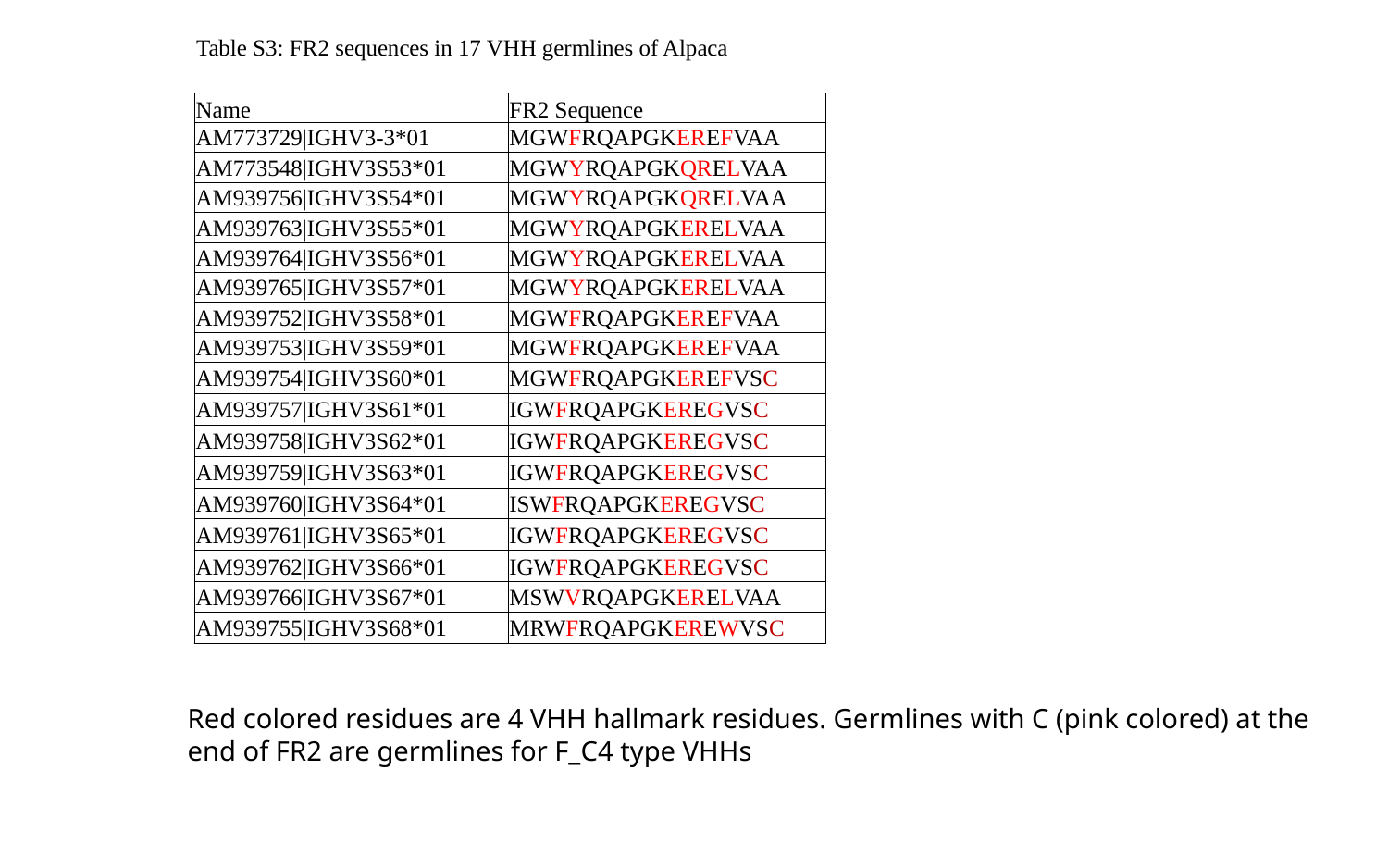

Table S3: FR2 sequences in 17 VHH germlines of Alpaca
| Name | FR2 Sequence |
| --- | --- |
| AM773729|IGHV3-3\*01 | MGWFRQAPGKEREFVAA |
| AM773548|IGHV3S53\*01 | MGWYRQAPGKQRELVAA |
| AM939756|IGHV3S54\*01 | MGWYRQAPGKQRELVAA |
| AM939763|IGHV3S55\*01 | MGWYRQAPGKERELVAA |
| AM939764|IGHV3S56\*01 | MGWYRQAPGKERELVAA |
| AM939765|IGHV3S57\*01 | MGWYRQAPGKERELVAA |
| AM939752|IGHV3S58\*01 | MGWFRQAPGKEREFVAA |
| AM939753|IGHV3S59\*01 | MGWFRQAPGKEREFVAA |
| AM939754|IGHV3S60\*01 | MGWFRQAPGKEREFVSC |
| AM939757|IGHV3S61\*01 | IGWFRQAPGKEREGVSC |
| AM939758|IGHV3S62\*01 | IGWFRQAPGKEREGVSC |
| AM939759|IGHV3S63\*01 | IGWFRQAPGKEREGVSC |
| AM939760|IGHV3S64\*01 | ISWFRQAPGKEREGVSC |
| AM939761|IGHV3S65\*01 | IGWFRQAPGKEREGVSC |
| AM939762|IGHV3S66\*01 | IGWFRQAPGKEREGVSC |
| AM939766|IGHV3S67\*01 | MSWVRQAPGKERELVAA |
| AM939755|IGHV3S68\*01 | MRWFRQAPGKEREWVSC |
Red colored residues are 4 VHH hallmark residues. Germlines with C (pink colored) at the end of FR2 are germlines for F_C4 type VHHs

## Slide 4
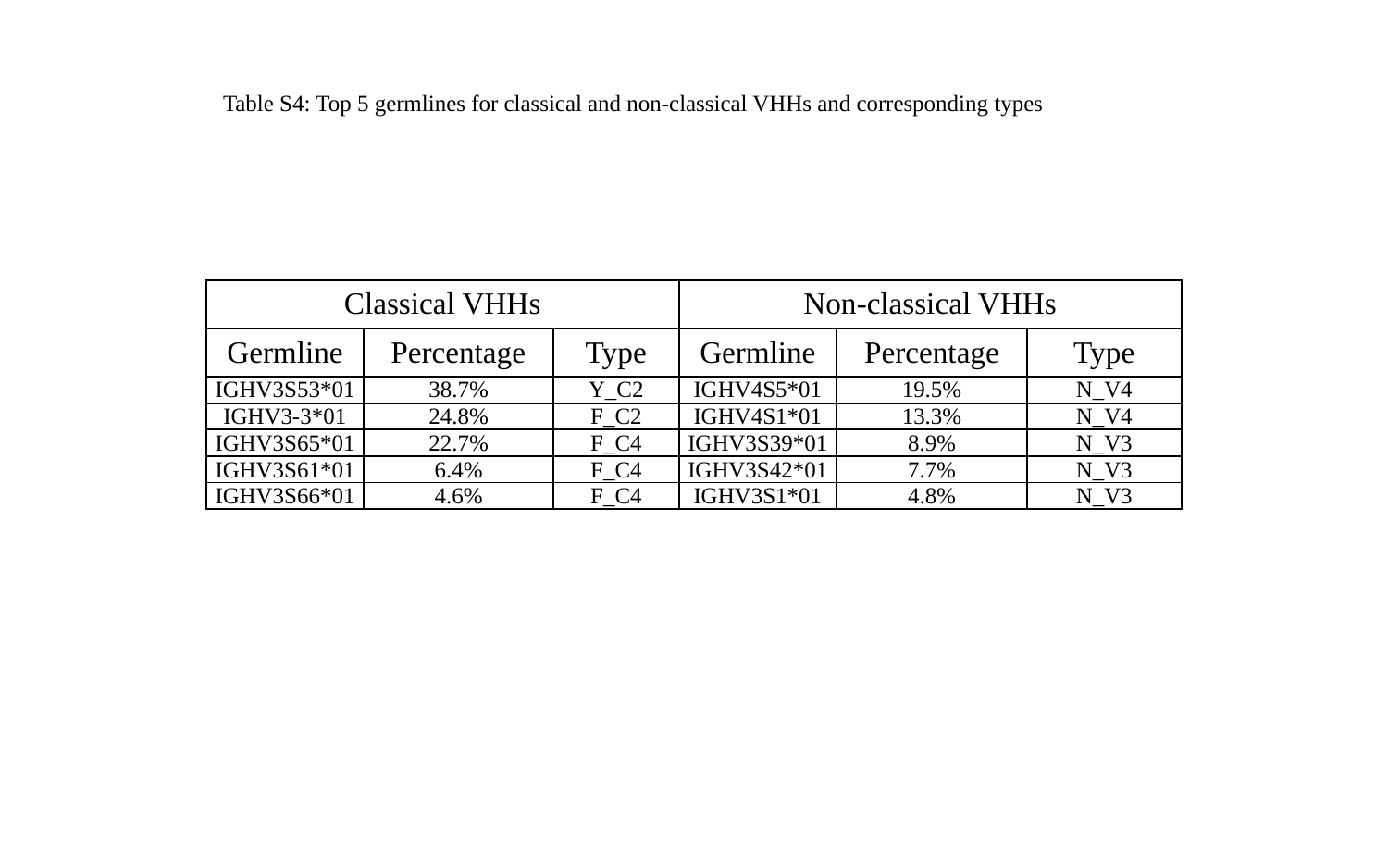

Table S4: Top 5 germlines for classical and non-classical VHHs and corresponding types
| Classical VHHs | | | Non-classical VHHs | | |
| --- | --- | --- | --- | --- | --- |
| Germline | Percentage | Type | Germline | Percentage | Type |
| IGHV3S53\*01 | 38.7% | Y\_C2 | IGHV4S5\*01 | 19.5% | N\_V4 |
| IGHV3-3\*01 | 24.8% | F\_C2 | IGHV4S1\*01 | 13.3% | N\_V4 |
| IGHV3S65\*01 | 22.7% | F\_C4 | IGHV3S39\*01 | 8.9% | N\_V3 |
| IGHV3S61\*01 | 6.4% | F\_C4 | IGHV3S42\*01 | 7.7% | N\_V3 |
| IGHV3S66\*01 | 4.6% | F\_C4 | IGHV3S1\*01 | 4.8% | N\_V3 |

## Slide 5
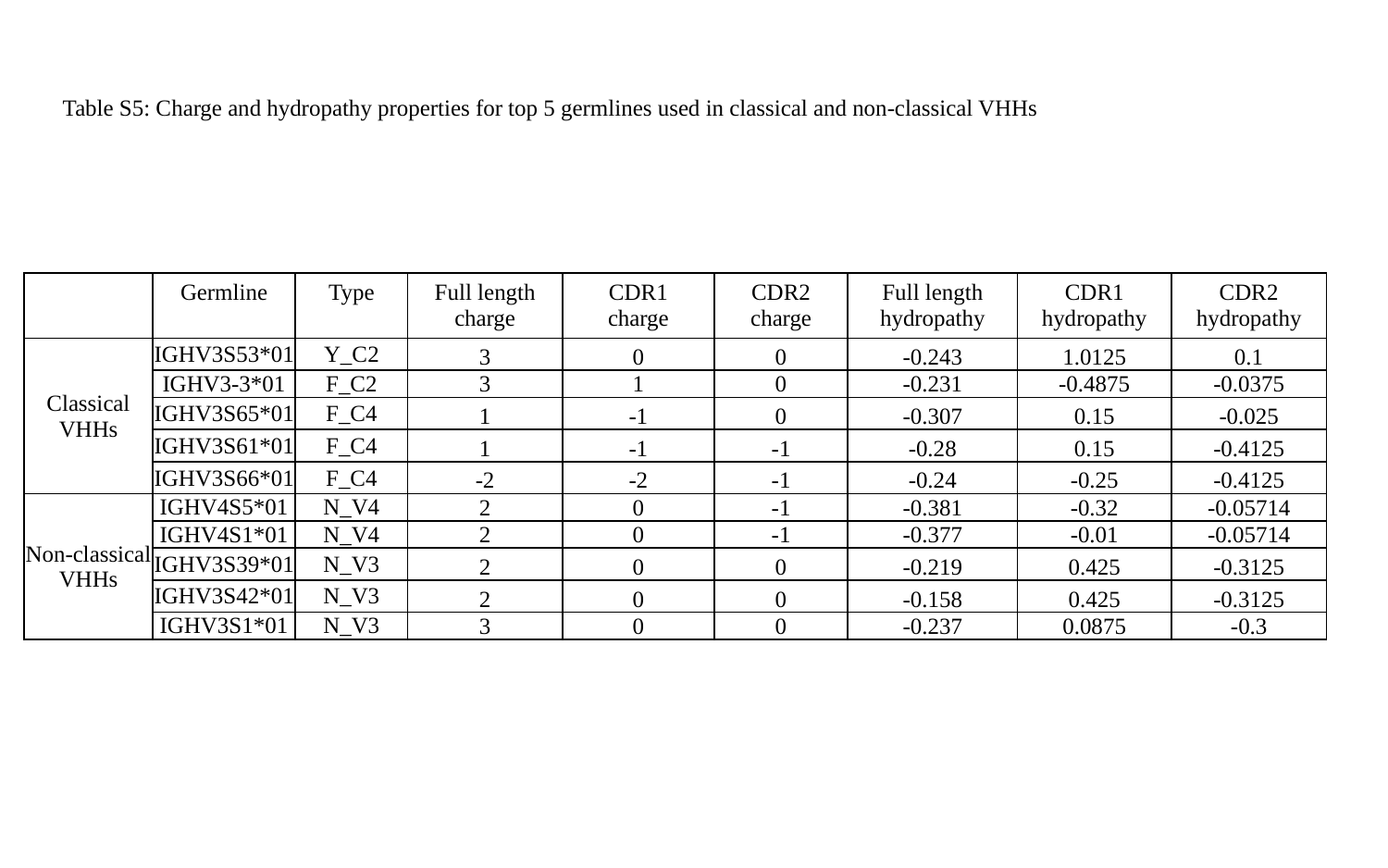

Table S5: Charge and hydropathy properties for top 5 germlines used in classical and non-classical VHHs
| | Germline | Type | Full length charge | CDR1 charge | CDR2 charge | Full length hydropathy | CDR1 hydropathy | CDR2 hydropathy |
| --- | --- | --- | --- | --- | --- | --- | --- | --- |
| Classical VHHs | IGHV3S53\*01 | Y\_C2 | 3 | 0 | 0 | -0.243 | 1.0125 | 0.1 |
| | IGHV3-3\*01 | F\_C2 | 3 | 1 | 0 | -0.231 | -0.4875 | -0.0375 |
| | IGHV3S65\*01 | F\_C4 | 1 | -1 | 0 | -0.307 | 0.15 | -0.025 |
| | IGHV3S61\*01 | F\_C4 | 1 | -1 | -1 | -0.28 | 0.15 | -0.4125 |
| | IGHV3S66\*01 | F\_C4 | -2 | -2 | -1 | -0.24 | -0.25 | -0.4125 |
| Non-classical VHHs | IGHV4S5\*01 | N\_V4 | 2 | 0 | -1 | -0.381 | -0.32 | -0.05714 |
| | IGHV4S1\*01 | N\_V4 | 2 | 0 | -1 | -0.377 | -0.01 | -0.05714 |
| | IGHV3S39\*01 | N\_V3 | 2 | 0 | 0 | -0.219 | 0.425 | -0.3125 |
| | IGHV3S42\*01 | N\_V3 | 2 | 0 | 0 | -0.158 | 0.425 | -0.3125 |
| | IGHV3S1\*01 | N\_V3 | 3 | 0 | 0 | -0.237 | 0.0875 | -0.3 |

## Slide 6
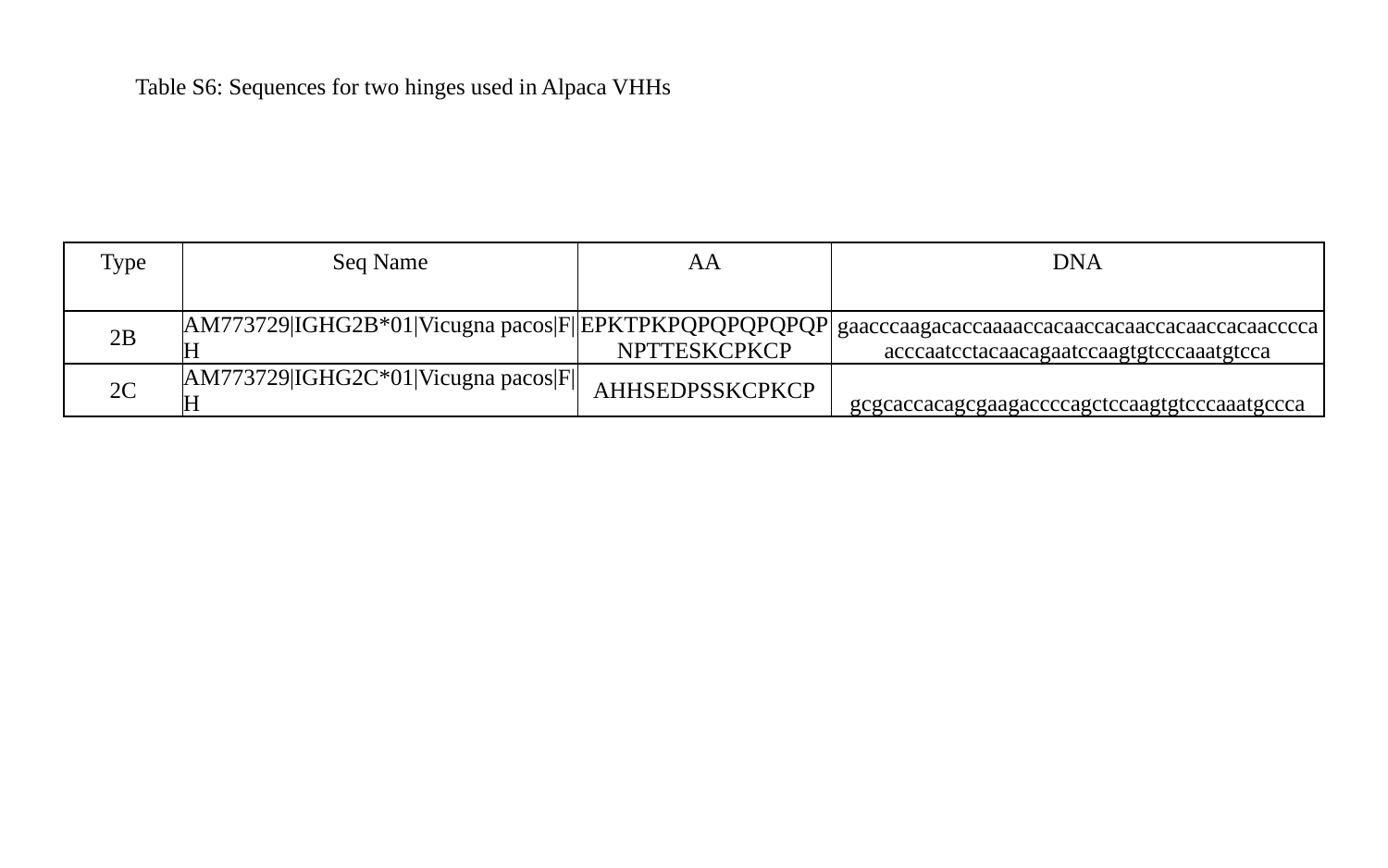

Table S6: Sequences for two hinges used in Alpaca VHHs
| Type | Seq Name | AA | DNA |
| --- | --- | --- | --- |
| 2B | AM773729|IGHG2B\*01|Vicugna pacos|F|H | EPKTPKPQPQPQPQPQPNPTTESKCPKCP | gaacccaagacaccaaaaccacaaccacaaccacaaccacaaccccaacccaatcctacaacagaatccaagtgtcccaaatgtcca |
| 2C | AM773729|IGHG2C\*01|Vicugna pacos|F|H | AHHSEDPSSKCPKCP | gcgcaccacagcgaagaccccagctccaagtgtcccaaatgccca |

## Slide 7
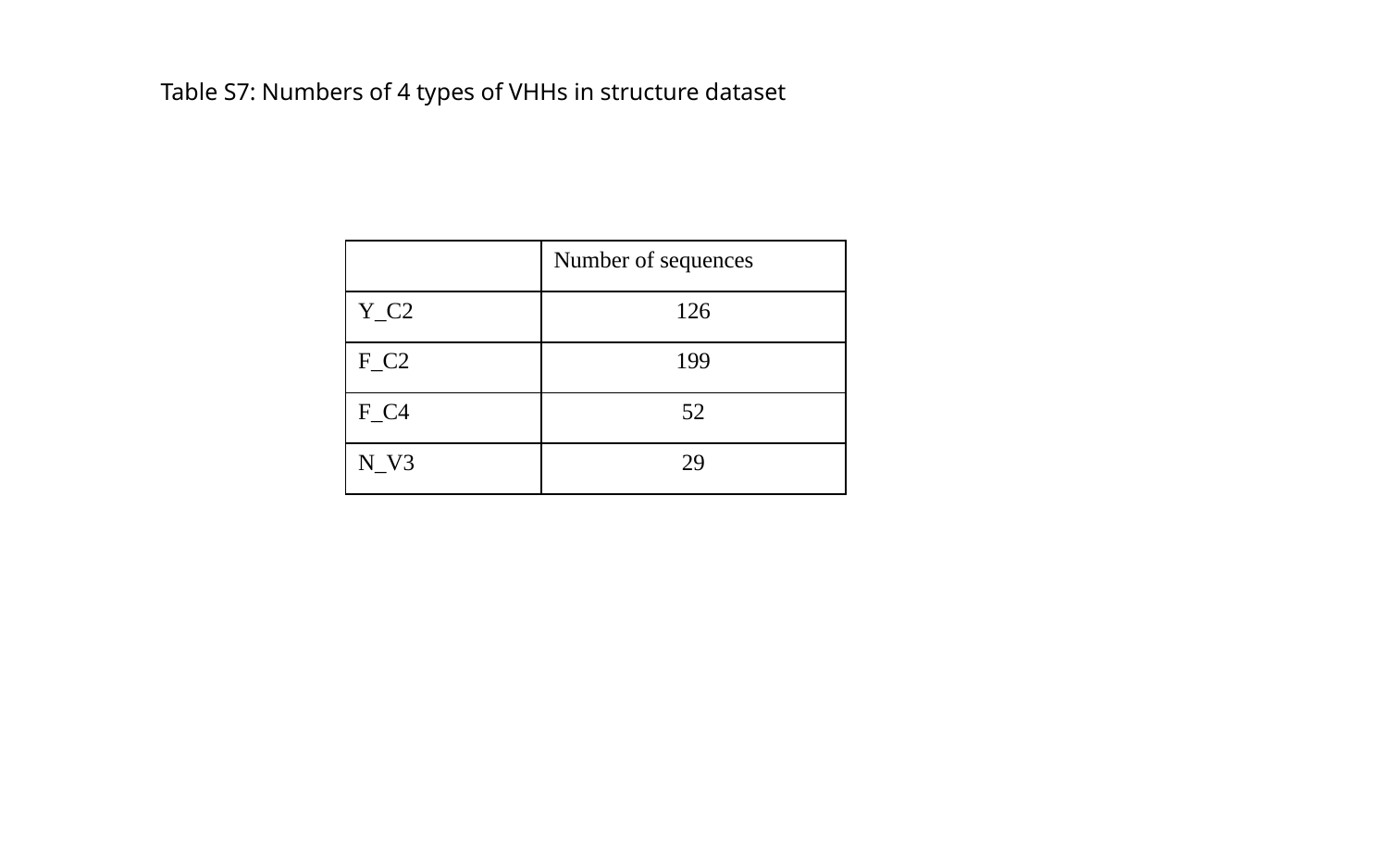

Table S7: Numbers of 4 types of VHHs in structure dataset
| | Number of sequences |
| --- | --- |
| Y\_C2 | 126 |
| F\_C2 | 199 |
| F\_C4 | 52 |
| N\_V3 | 29 |

## Slide 8
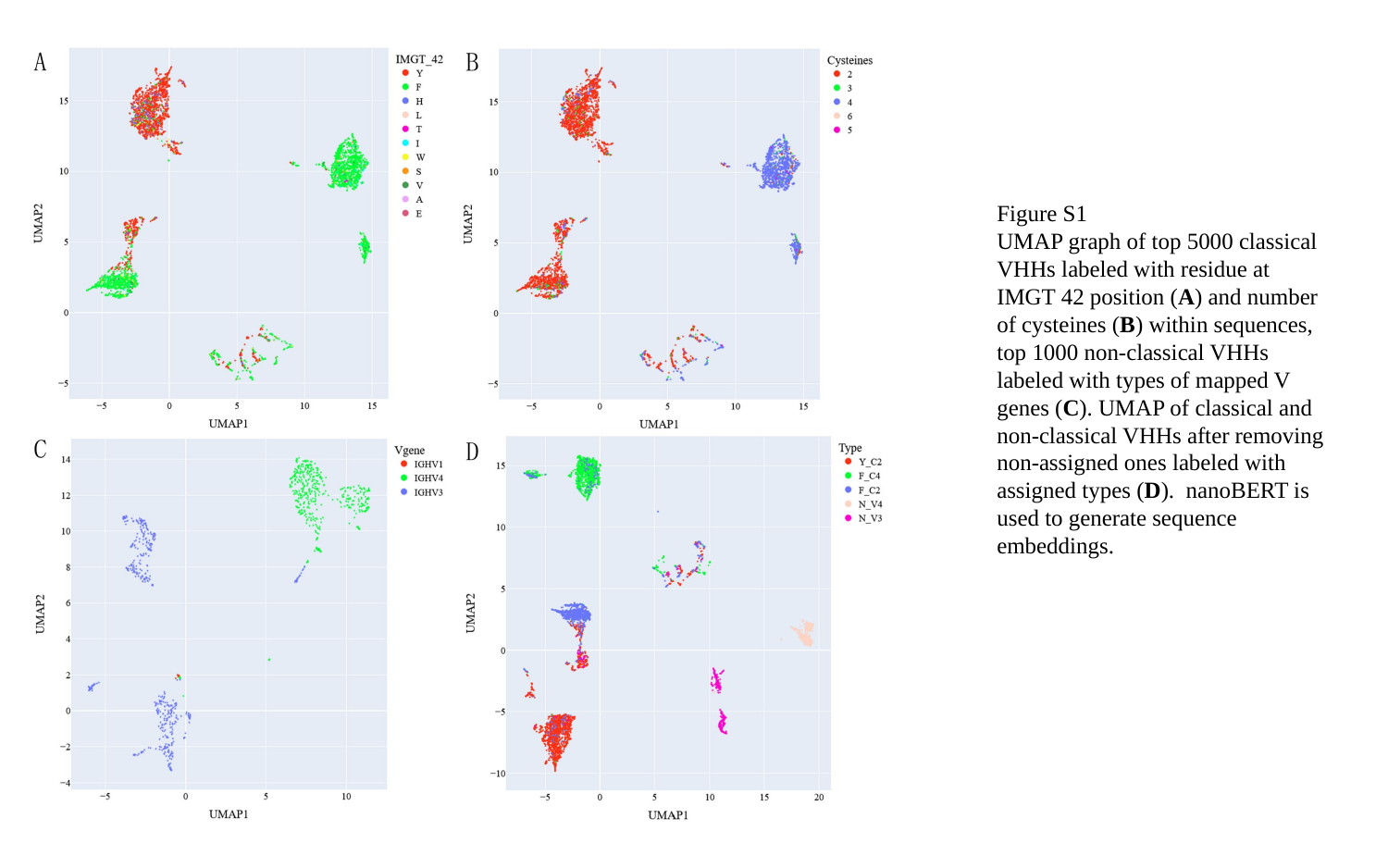

Figure S1
UMAP graph of top 5000 classical VHHs labeled with residue at IMGT 42 position (A) and number of cysteines (B) within sequences, top 1000 non-classical VHHs labeled with types of mapped V genes (C). UMAP of classical and non-classical VHHs after removing non-assigned ones labeled with assigned types (D). nanoBERT is used to generate sequence embeddings.

## Slide 9
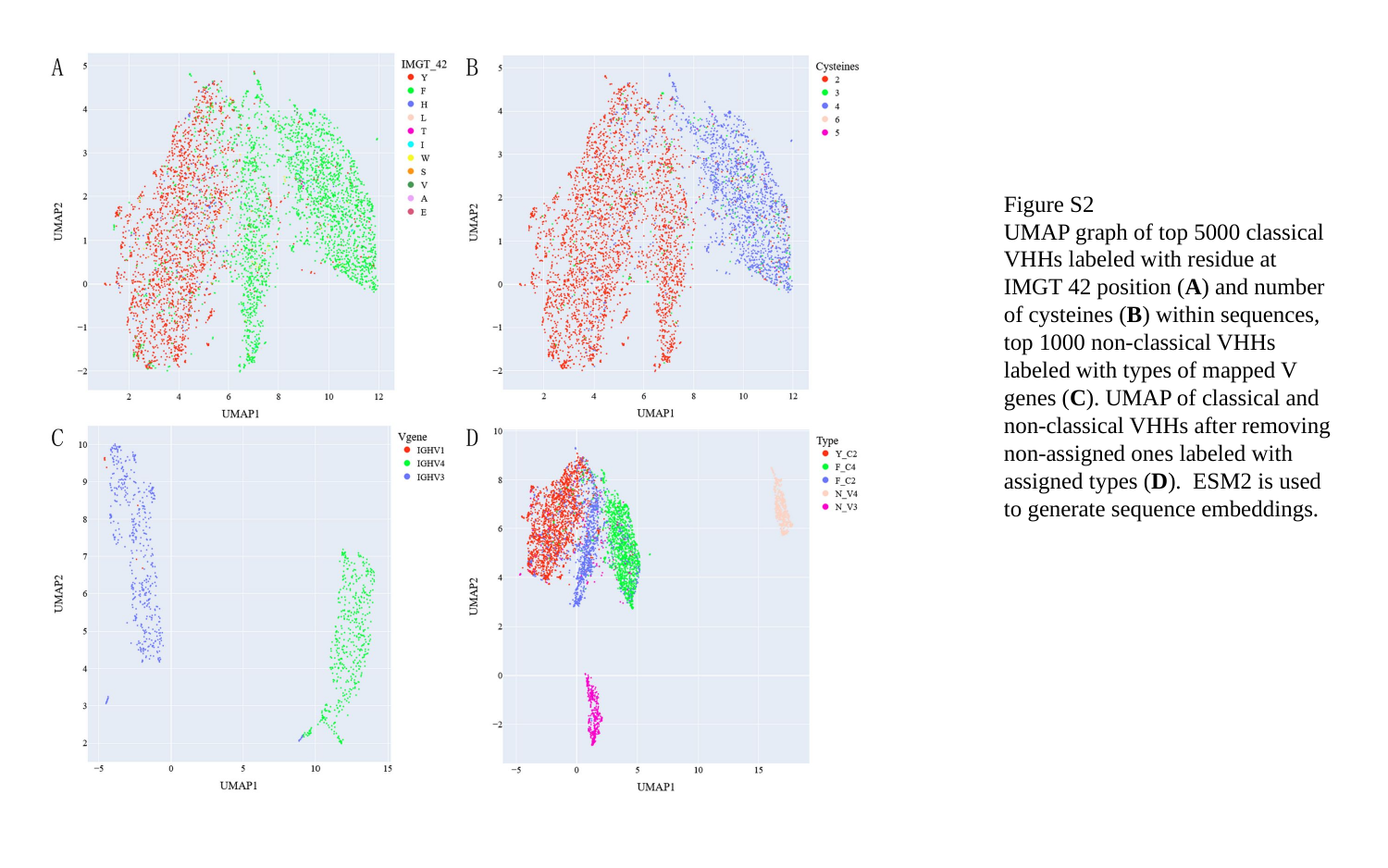

Figure S2
UMAP graph of top 5000 classical VHHs labeled with residue at IMGT 42 position (A) and number of cysteines (B) within sequences, top 1000 non-classical VHHs labeled with types of mapped V genes (C). UMAP of classical and non-classical VHHs after removing non-assigned ones labeled with assigned types (D). ESM2 is used to generate sequence embeddings.

## Slide 10
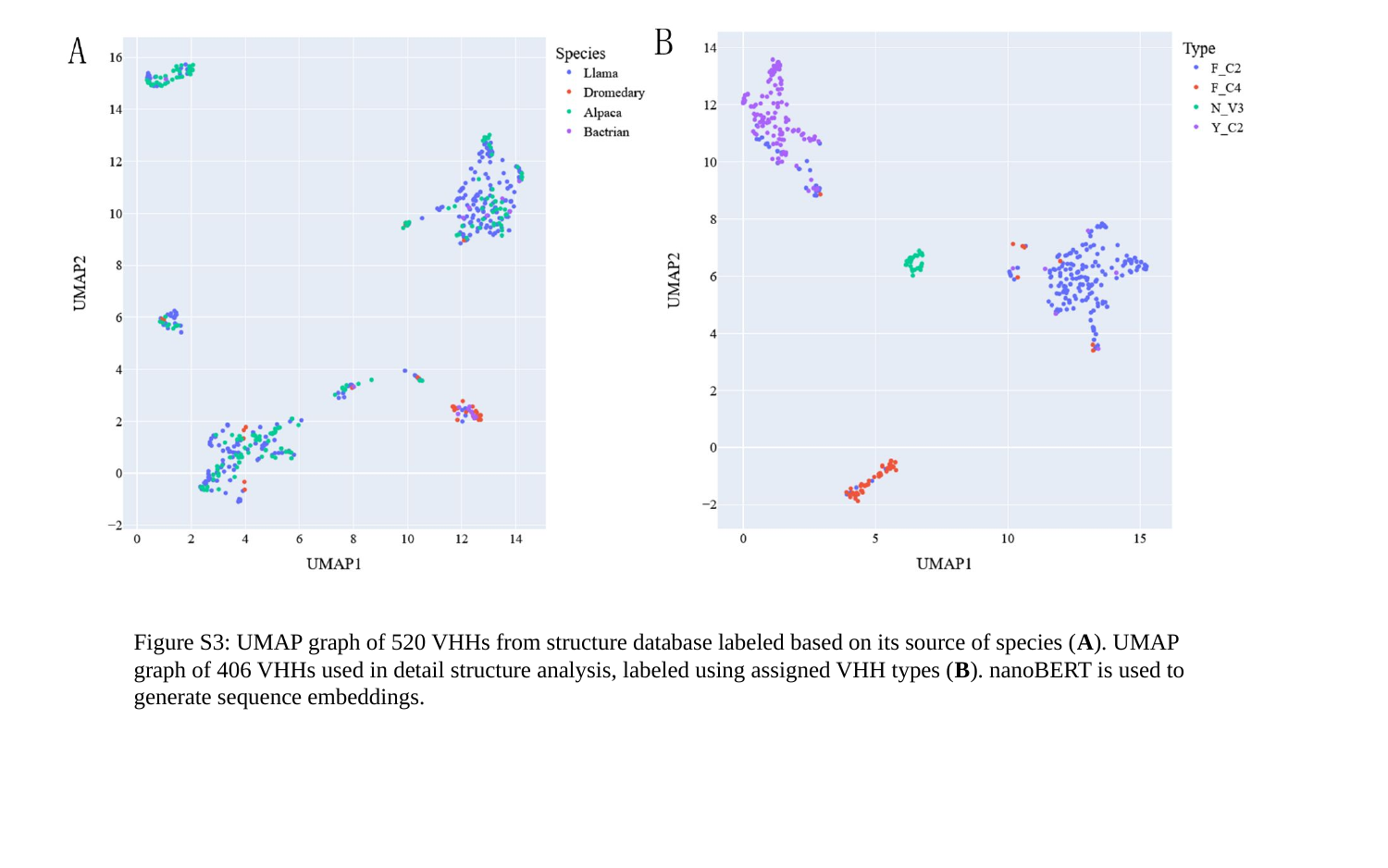

Figure S3: UMAP graph of 520 VHHs from structure database labeled based on its source of species (A). UMAP graph of 406 VHHs used in detail structure analysis, labeled using assigned VHH types (B). nanoBERT is used to generate sequence embeddings.

## Slide 11
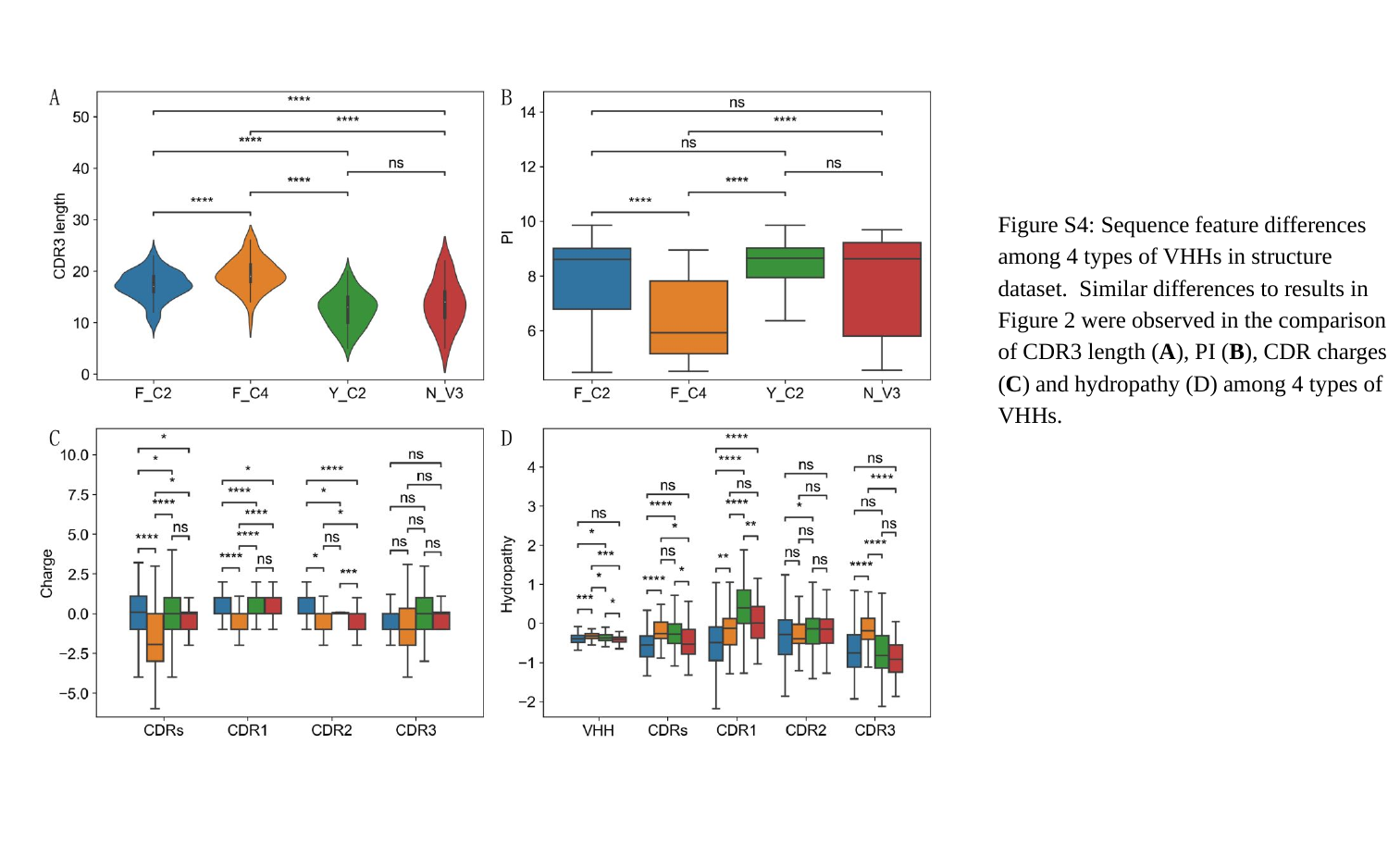

Figure S4: Sequence feature differences among 4 types of VHHs in structure dataset. Similar differences to results in Figure 2 were observed in the comparison of CDR3 length (A), PI (B), CDR charges (C) and hydropathy (D) among 4 types of VHHs.

## Slide 12
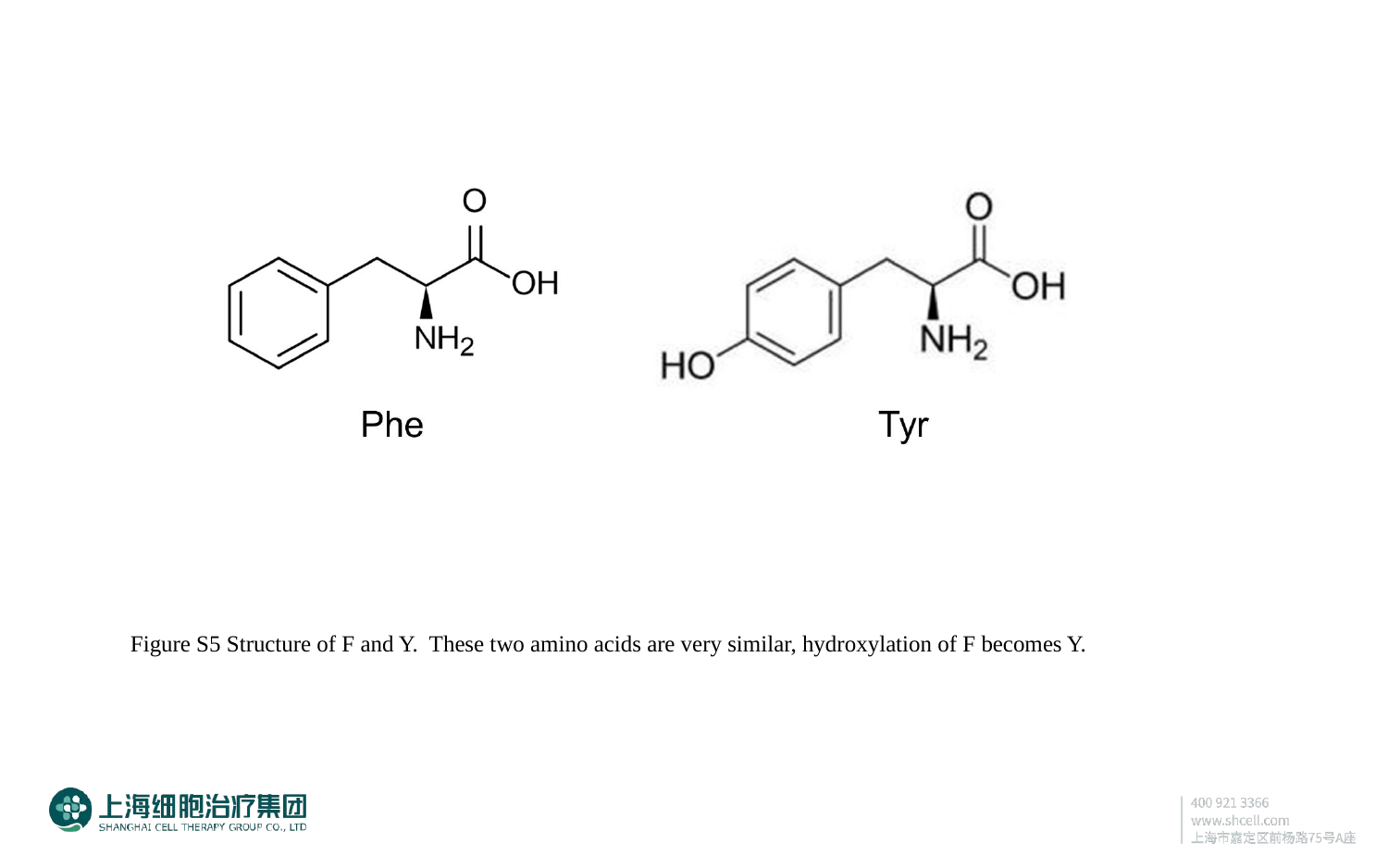

# Figure S5 Structure of F and Y. These two amino acids are very similar, hydroxylation of F becomes Y.

## Slide 13
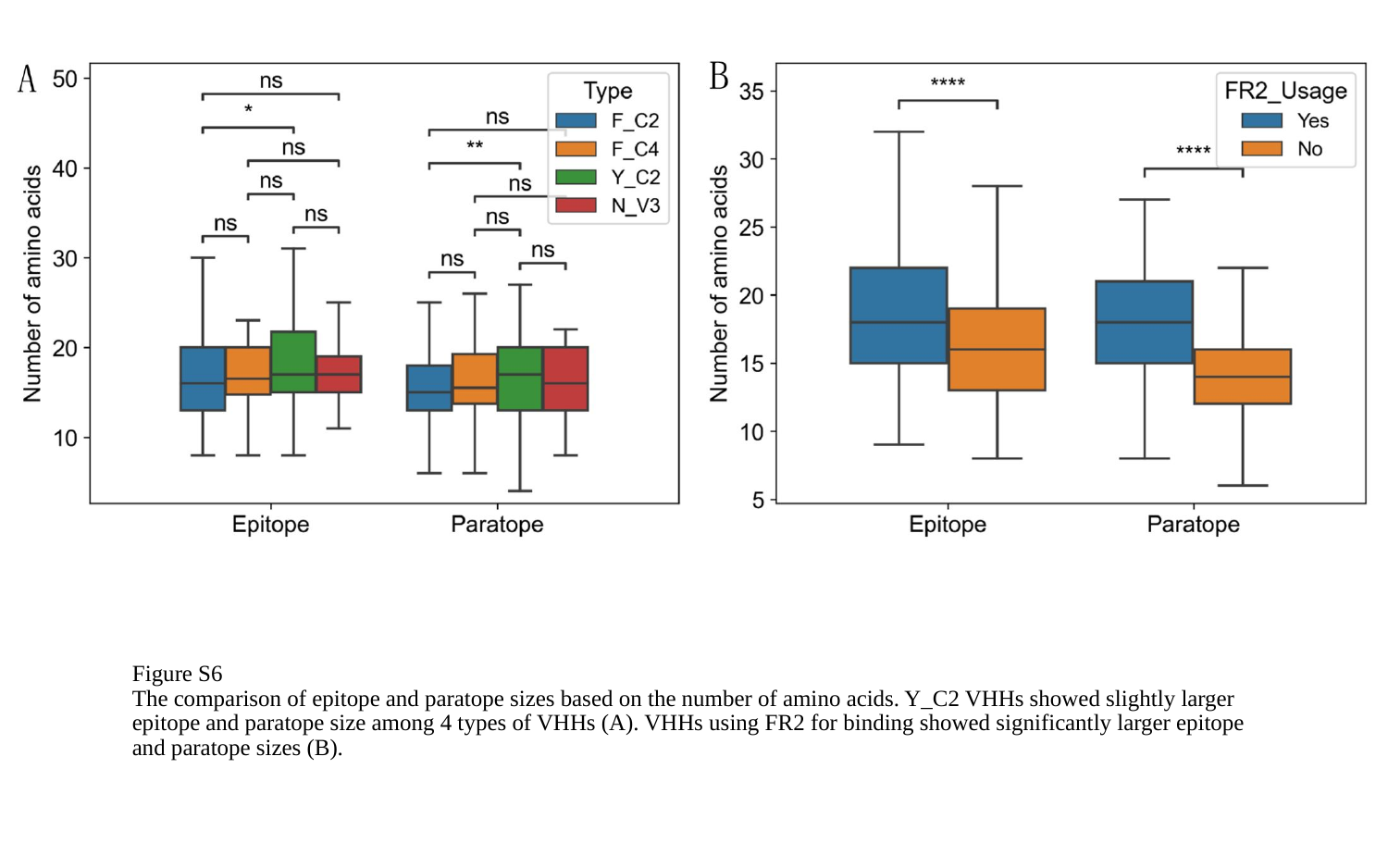

Figure S6
The comparison of epitope and paratope sizes based on the number of amino acids. Y_C2 VHHs showed slightly larger epitope and paratope size among 4 types of VHHs (A). VHHs using FR2 for binding showed significantly larger epitope and paratope sizes (B).

## Slide 14
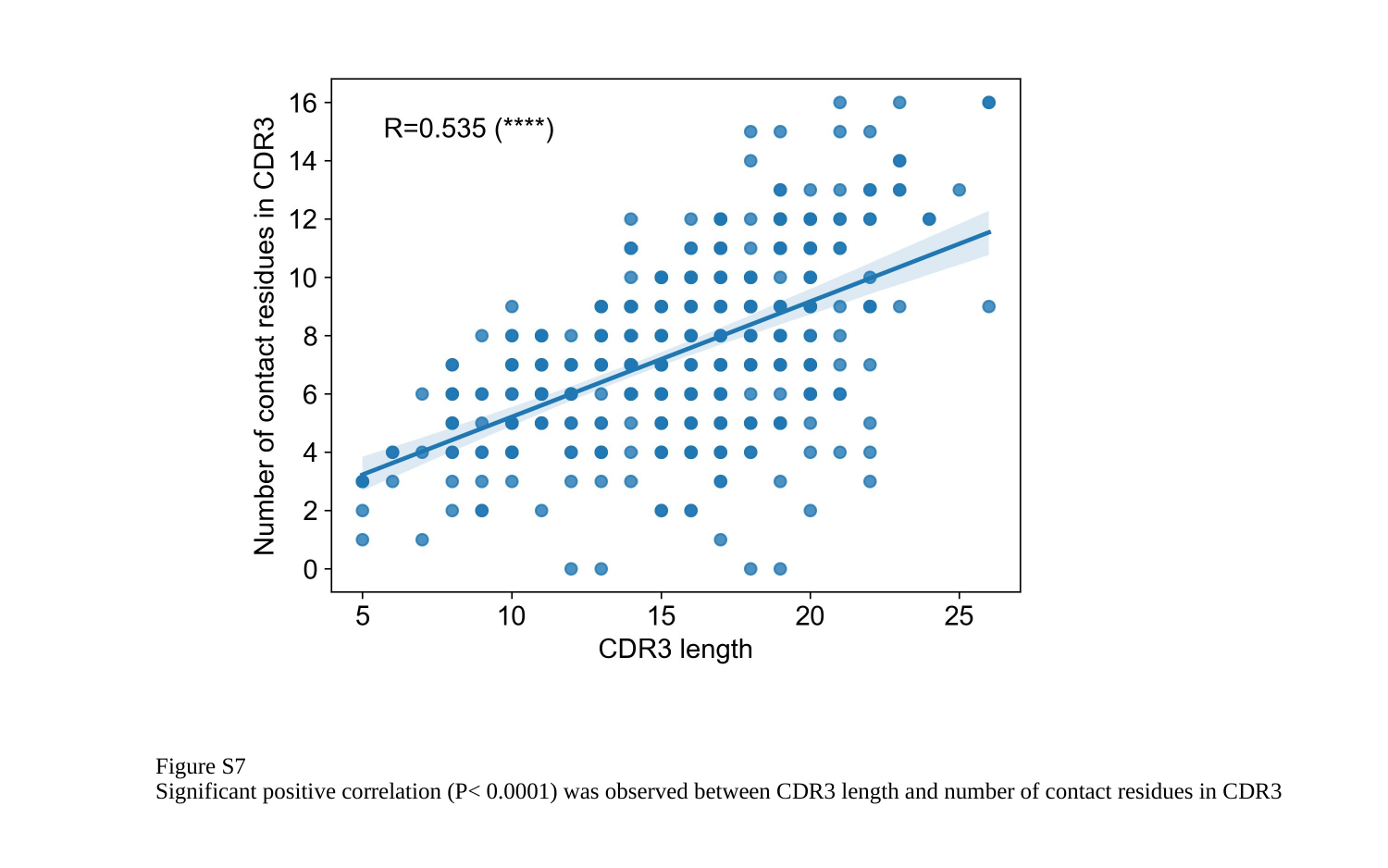

Figure S7
Significant positive correlation (P< 0.0001) was observed between CDR3 length and number of contact residues in CDR3

## Slide 15
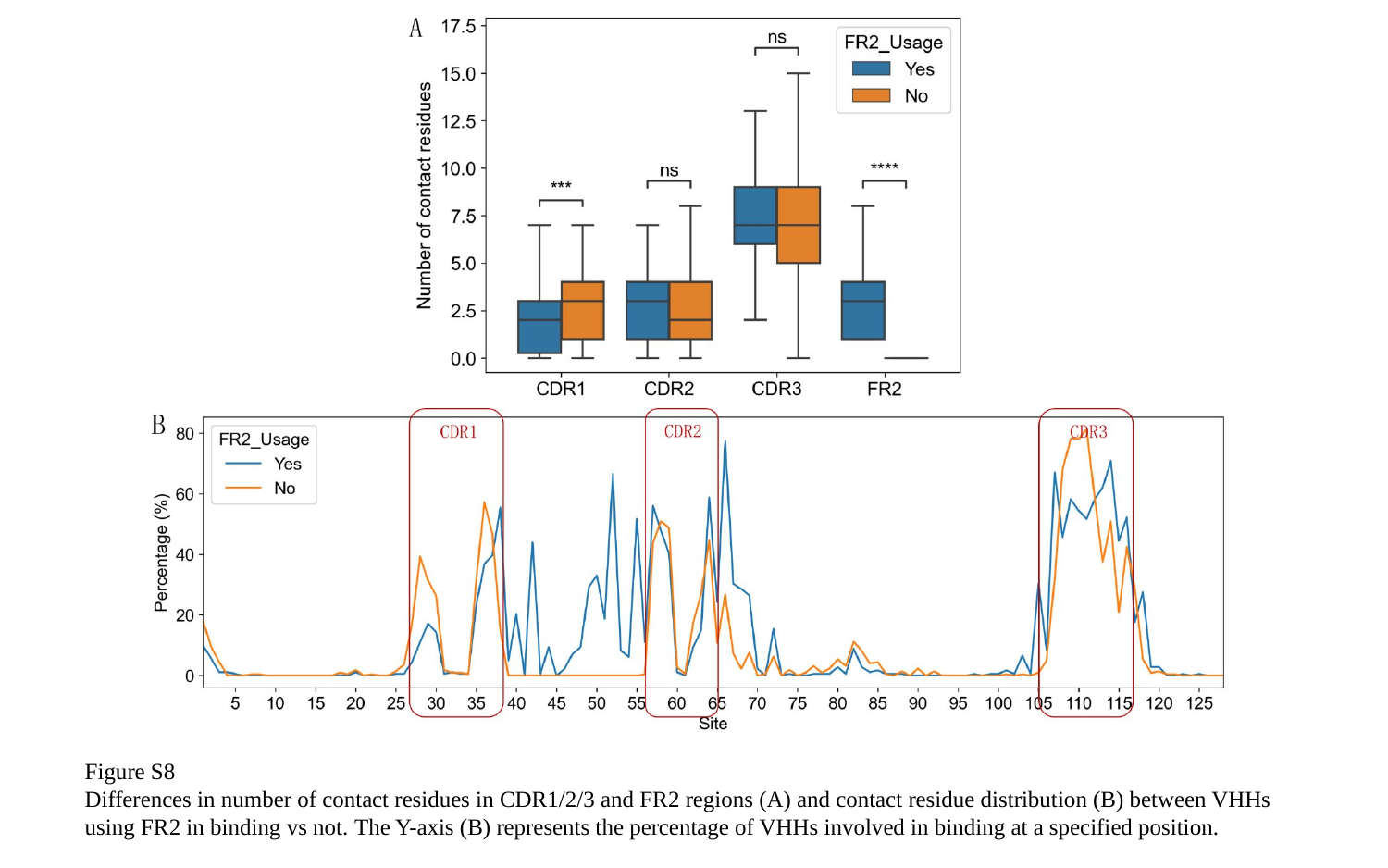

Figure S8
Differences in number of contact residues in CDR1/2/3 and FR2 regions (A) and contact residue distribution (B) between VHHs using FR2 in binding vs not. The Y-axis (B) represents the percentage of VHHs involved in binding at a specified position.

## Slide 16
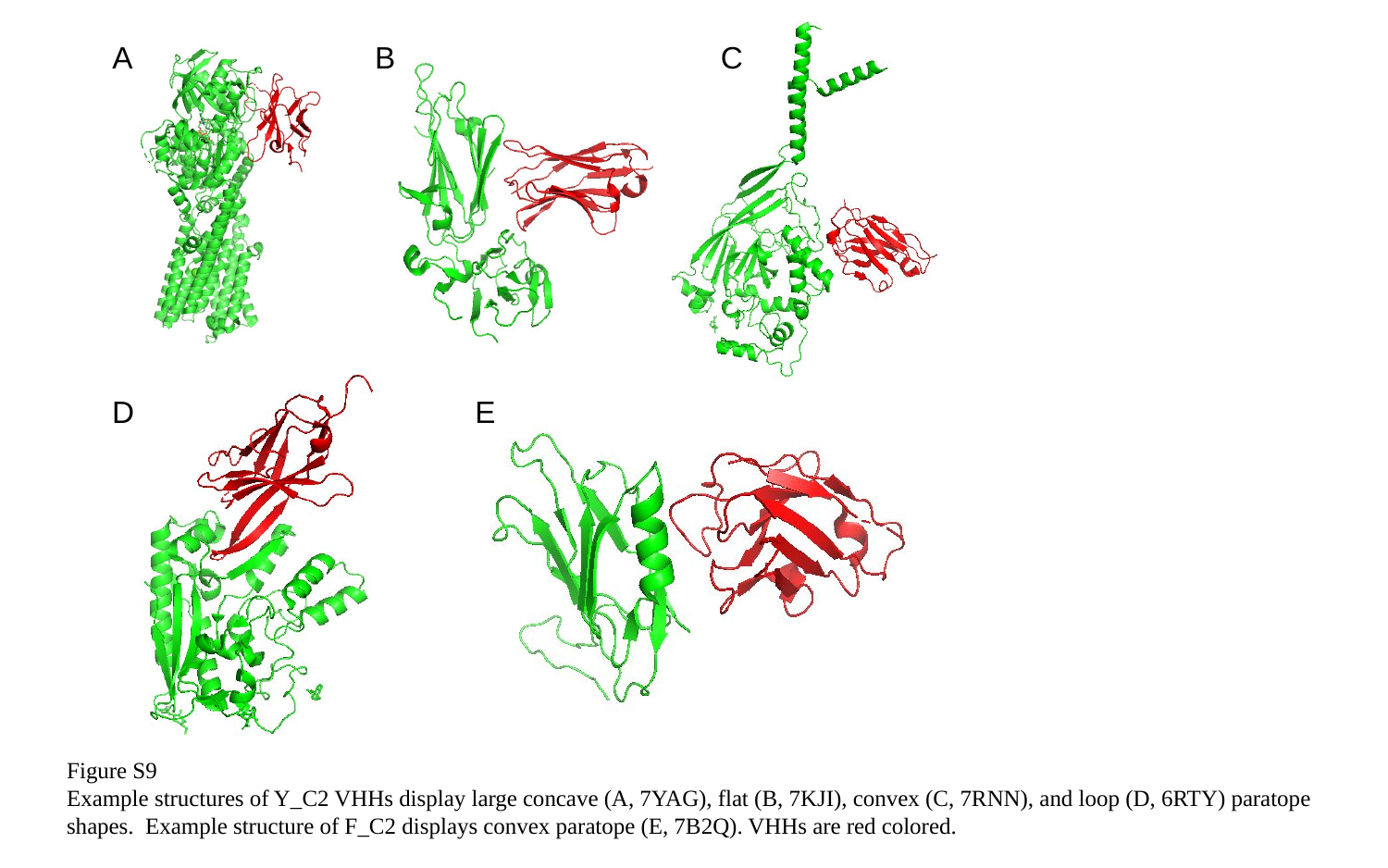

A
B
C
D
E
Figure S9
Example structures of Y_C2 VHHs display large concave (A, 7YAG), flat (B, 7KJI), convex (C, 7RNN), and loop (D, 6RTY) paratope shapes. Example structure of F_C2 displays convex paratope (E, 7B2Q). VHHs are red colored.
